# Light Concentration by Metal-Dielectric Micro-Resonators for SERS Sensing

**DOI:** 10.3390/ma12010103

**Published:** 2018-12-29

**Authors:** Andrey K. Sarychev, Andrey Ivanov, Andrey Lagarkov, Grégory Barbillon

**Affiliations:** 1 Institute for Theoretical and Applied Electrodynamics, Russian Academy of Sciences, 125412 Moscow, Russia; sarychev_andrey@yahoo.com (A.K.S.); lag@dol.ru (A.L.); 2 EPF-Ecole d’Ingenieurs, 3 bis rue Lakanal, 92330 Sceaux, France; gregory.barbillon@epf.fr

**Keywords:** metal-dielectric resonance, plasmon, metasurface, nanoparticles, sensing, surface-enhanced Raman scattering (SERS)

## Abstract

Metal-dielectric micro/nano-composites have surface plasmon resonances in visible and near-infrared domains. Excitation of coupled metal-dielectric resonances is also important. These different resonances can allow enhancement of the electromagnetic field at a subwavelength scale. Hybrid plasmonic structures act as optical antennae by concentrating large electromagnetic energy in micro- and nano-scales. Plasmonic structures are proposed for various applications such as optical filters, investigation of quantum electrodynamics effects, solar energy concentration, magnetic recording, nanolasing, medical imaging and biodetection, surface-enhanced Raman scattering (SERS), and optical super-resolution microscopy. We present the review of recent achievements in experimental and theoretical studies of metal-dielectric micro and nano antennae that are important for fundamental and applied research. The main impact is application of metal-dielectric optical antennae for the efficient SERS sensing.

## 1. Introduction

In the short review, we present recent results in plasmonics of metal-dielectric composites and metasurfaces. Optical properties of metal nanoparticles are intensively studied for more than one hundred years [1,2,3]. Surface plasmons can confine the electromagnetic (EM) field to a nanoscale (hotspots), which can enhance greatly this EM field. The modern technology allows for designing and producing metal nanostructures of different shapes and sizes. The specially designed metal nanostructures serve as optical antennae, which opens exciting opportunities in fundamental physics studies, but also in plasmonic applications such as optical signal processing on a nanoscale, medical imaging and biodetection, optical super-resolution microscopy [4], magnetic recording assisted by heat [5,6], quantum electrodynamics studies [7], nanolasing, and solar energy concentrators [8]. A strongly amplified electromagnetic field can be generated by disordered metal-dielectric composites in a broad spectral range [9]. Periodically ordered nanostructures enhance local EM field at selected frequencies [10,11,12,13,14,15,16,17,18,19,20,21,22,23]. Plasmon modes propagating in a chain of metallic nanoparticles, where the particle radius *a* is relatively equal to the distance δ between particles, are precisely investigated in [24]. Concerning these modes, the near-field interaction allows the jump of the dipolar excitation between each particle. EM field is confined in the chain vicinity. Guided modes of the nanoparticle chain, which propagate in the domain a≫λ, were studied in [25,26,27]. These modes are similar to dipoles modes propagating around an optically thin cylinder. Scattering/diffracting experiments of an EM wave with nanorod periodic arrays were showed in [28,29]. Recently, the wave propagation along metallic nanorods is an attractive issue. Indeed, the negative refraction can be realized with these types of systems [30,31,32,33]. Fascinating optical effects such as Doppler shift, Cherenkov-Vavilov radiation, light pressure and Magnus effects are anomalous/inverse in negative refractive materials [34,35,36,37]. Stacked nanorods are proposed for the microwave and optical superresolution imaging [38,39,40,41,42,43,44]. The EM field mappings for the metal nanoparticles and nanoshells in a close-packed configuration were demonstrated in [45] and [46,47], respectively. In [11], an metallic nanocylinder array of which the cylinders are very close is investigated. At the resonance, the EM field is significantly improved in the gaps between metallic nanocylinders. This enhancement is due to the excitation surface plasmons (SPs) in the gap between almost touching (“kissing”) metal cylinders. Electromagnetic resonators can be formed by dielectric optical microcavities. The optical microcavities can confine the light at the micro/nanoscale. Optical resonators can be applied to different domains such as resonators enabling data transmission by using optical fibers. Furthermore, they can be used for obtaining narrow spot-size laser beams for the reading and writing of CD/DVD. Microcavities can force an atom or quantum dot to spontaneously producing a photon in a given direction. In quantum optical devices, dissipation can be overcome in order to potentially obtain a quantum entanglement of the matter and radiation (see Refs. [7,48,49]). In addition, the optical behavior of metals is strongly damped due to important losses as interband transitions and intraband transitions due to impurity scattering in solids and additional surface scattering. The losses result in the heat degradation of metal nanoparticles. The degradation reaches the highest value in maxima of the local electric field. Another issue for metalic nanoparticles is the chemical instability. Plasmonic nanostructures of gold are well-known for being the most chemically stable. Unfortunately, large losses of gold due to interband transitions occur in the visible domain for the wavelengths λ<600 nm [50]. The blue loss in gold gives the yellow color. The optical properties of metals described previously have a negative effect on the sensor efficiency. For all the low loss dielectrics, electromagnetic resonances can be obtained by light excitation. However, the quality factor *Q* varies with the type of the EM modes and, for some of them, they have losses even for huge resonators [51]. For instance, the whispering-gallery modes (WGM) excited in dielectric resonators made of silica, ${\mathrm{CaF}}_{2}$, ${\mathrm{MgF}}_{2}$, GaN or GaAs have large *Q*-factors. The shape of the WGM resonators is usually a torus, disk or sphere. The values of the *Q*-factor for a WGM resonator can achieve $10^{7}$ and $10^{9}$ [52,53,54,55,56,57]. These WGM resonators can allow the realization of filters, modulators, sensors or lasers. Due to a long lifetime of a WGM, a single molecule or virus can be detected on the cavity surface [58,59]. In this last decade, the concentration of electric and magnetic fields in the dielectric micro-structures has had a great amount of attention [60,61]. For two resonant dielectric spheres, the electric field located within the gap of these spheres was enhanced and demonstrated in [62,63]. A Yagi–Uda antenna can be composed of a chain of six dielectric nanoparticles [64]. This latter can significantly enhance the radiation of a dipole placed between these particles. Therefore, the antenna can confine the incident light in this same location. The enhancement of light was obtained with a ring of plasmonic nanoparticles coupled to a dielectric micro-resonator [65]. The surface plasmon resonance of the nanoparticle ring enables the EM field enhancement close to the dielectric micro-resonator surface. On the contrary, the electric field is significantly less important in the case of a dielectric resonator and when the metallic nanoparticles are spatially separated [66]. The light propagation in dielectric metamaterials is discussed in the review paper [67]. For instance, the EM wave can be confined within a nanoscopic volume with a dielectric waveguide having an anisotropic cladding [68]. The electric field confinement between dielectric rectangular resonators was demonstrated by [69]. The super resolution of resonant microstructures can be achieved by a dielectric microsphere (see [70] and references therein).

The optical nonlinearity can be enhanced by using a magnetic resonance obtained with four closely packed dielectric disks [71]. Kim et al. have showed an effective absorption of the EM field in periodic semiconductor metafilms for solar cells (see [72]). Some groups have demonstrated a strong electric field and SERS enhancement for periodic metafilms made of rectangular dielectric bars [12,16]. Sharp minima in the reflectance of the dielectric metasurface can be achieved in the microwave and optical domains. Distributed dielectric resonances in randomly cracked ceria metafilms were also considered [13]. Dielectric metamaterials can be used for biosensing (see Refs. [73,74,75]). All-dielectric and metal-dielectric 2D and 3D light concentrators were investigated in [16,17]. It was shown that plasmon and dielectric resonances can be independently managed, i.e., the frequencies of the resonances can be independently tuned by varying of the shape, arrangement and the nature of the metals and dielectrics. Thus, the enhancement of SERS signal is additionally increased by combining plasmonic and dielectric resonators.

## 2. Plasmon Resonance and Field Enhancement

**Figure 1:**
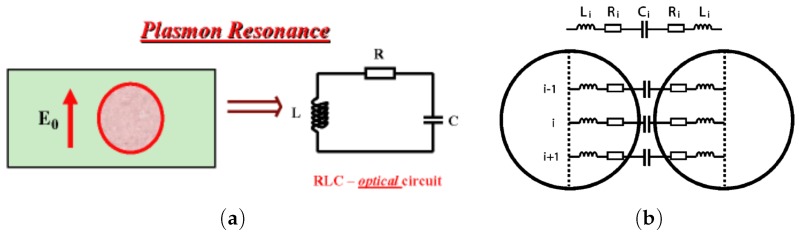
(**a**) L-C-R contour mapping of a plasmonic nanoparticle resonance. Plasmonic nanoparticle is represented by the Inductance (L) and resistance (R), and the surrounding medium by the capacitor (C); (**b**) plasmonic response depending on the frequency described as an L-C-R circuit array, reprinted with permission from [45], Copyright 2004 American Chemical Society, and from [76], Copyright 2007 World Scientific Publishing Co. Pte. Ltd.

**Figure 2:**
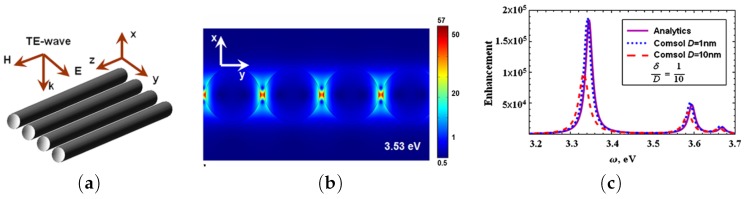
(**a**) scheme of the TE-wave propagation for an array of closely packed nanocylinders; (**b**) electric field mapping of surface plasmon resonances in an array of Ag cylinders with the following parameters: ω=3.53eV, cylinder radius a=5 nm, interparticle distance δ=1nm; (**c**) comparison of analytical (purple line) and numerical (blue and red dashed lines) enhancements $|E_{m}/E_{0}{|}^{2}$ of the electromagnetic (EM) field in the middle of dimer. The ratio δ/D is equal to 0.1, the nanorod diameter for COMSOL simulations is D=2a=10nm or 1nm, and $E_{0}$ is amplitude of incident light, reprinted by permission from Springer Nature: Springer Nature, Applied Physics A: Materials Science and Processing [11], Copyright 2012.

Plasmon resonance can be explained in terms of L-C-R circuit [45,76]. Knowing the negativity of the metal permittivity in the optical frequency range, the optical electric current inside a metal nanoparticle is opposite to the direction of the displacement current outside the particle. Therefore, the metallic particle can be modeled as an inductance L. The excitation of the L-C-R contour models the interaction between the plasmonic nanoparticle and the EM field (Figure 1a). Here, the plasmonic nanoparticle is thus represented by the inductance L of which small losses are modeled by resistance R, and the surrounding medium is modeled by the capacitance C. Thus, the resonance in the L-C-R circuit models the plasmon resonance of a single metallic nanoparticle. An array of L-C-R circuits’ models the EM coupling between two adjoined plasmonic nanoparticles. It is quite evident from the lumped circuit for the almost touching particles (Figure 1b) that there exists a longitudinal resonance when all the gap capacitances operate at the same phase. In addition, there are transverse modes propagating along a L-C chain, which represents the interparticle gap [11]. A narrow gap between metallic nanoparticles can be considered as a metal-dielectric waveguide, where the standing plasmon waves are excited (see, e.g., Figure 2).

The L-C-R model (Figure 1b) shows that the frequencies of the collective plasmon resonances for nanoparticle arrays decrease (corresponding to high values of L and C) with increasing of diameter-spacing ratio a/δ. In addition, the model agrees with the broadening of the plasmon bandwidth that occurs with red-shifting of resonance frequencies. The L-C-R model gives a description of the EM field enhancement depending on a,δ and $\epsilon_{m}$. The electric field mapping and field enhancement in a two-dimensional (2D) periodic array of infinite metallic cylinders are shown in Figure 2. The analytical expression for the EM field enhancement in the gap between the nanorods gives the results that are very similar to computer simulations [11]. The EM field mapping shows excitation of multiple plasmon resonances. In a system of nanorods, SPs are strongly localized between the rods, and a large enhancement of the local EM field is achieved. The resonance frequencies are given by this simple equation:

(1) Re[εm(ωq)]=−εdγ2q+1γ2q−1,

where q=1,2,3,…, $\varepsilon_{d}$ is the permittivity of the host medium, the parameters are $\gamma =l/a+\sqrt{1+{\left(l/a\right)}^{2}}$, $l=\sqrt{\delta (a+\delta /4)}$, and δ is the distance between cylinders of the radius *a*. For the closely packed cylinders, when δ≪a the characteristic scale $l\approx \sqrt{a\delta }$ and the parameter γ≈1+l/a is close to unity. The position of the resonances can be controlled by adjusting the diameter-spacing ratio. The above defined characteristic length *l* is those of the effective plasmonic waveguide between nanocylinders. The electric field between the cylinders can be found by conform mapping and by using new coordinates $u+iv=\ln\left[\left(il+z\right)/\left(il-z\right)\right]$ instead of the original coordinates z=x+iy to solve the Laplace equation. Thus, the field enhancement in the middle point between the cylinders (x=y=0) is given by the Equation:

(2) $${\left|E/E_{0}\right|}^{2}={\left|1+8\sum_{q=1}^{\infty }{\left(-1\right)}^{q}\frac{q\;\alpha_{m}}{\gamma^{2q}+\alpha_{m}}\right|}^{2},$$

where $E_{0}$ corresponds to the incident field amplitude and $\alpha_{m}=\left(\epsilon_{d}-\epsilon_{m}\right)/\left(\epsilon_{d}+\epsilon_{m}\right)$ is proportional to the polarizability of a metallic nanocylinder. The electric field is the sum of the resonance terms. Recall that the permittivity of metals is almost negative in the optical spectral domain of which the imaginary part is small $\varepsilon_{m}^{\prime \prime }\ll \left|\varepsilon_{m}^{\prime }\right|$. The denominators in the sum in Equation (2) almost vanishes at the resonance frequencies given by Equation (1). The maximum of the electric field at the *q*-th resonance estimates as

(3) $${\left|E_{q}/E_{0}\right|}^{2}≃256\;q^{2}\frac{\gamma^{4q}\epsilon_{d}^{2}}{\epsilon_{m}^{\prime \prime }{\left(\omega_{q}\right)}^{2}{\left(\gamma^{2q}-1\right)}^{4}},$$

where $E_{q}$ is the *q*-th resonance electric field in the middlepoint between the cylinders, $\epsilon_{m}^{\prime \prime }\left(\omega_{q}\right)$ is the value of the imaginary part of the metal permittivity at the resonance frequency $\omega_{q}$. The resonances are well-seen in Figure 2. The field enhancement ${\left|E\left(x,y\right)/E_{0}\right|}^{2}$ in the system of the adjoined nanocylinders can be as large as $10^{5}$ or even larger [11]. We note below that SERS is proportional to ${\left|E\left(x,y\right)/E_{0}\right|}^{4}$, and, therefore, is indeed huge in the system of the metal nanocylinders.

**Figure 3:**
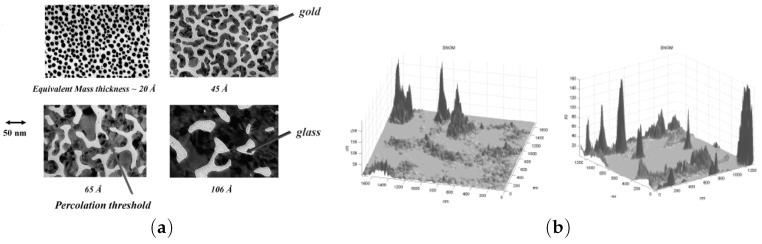
(**a**) gold/glass composite for four metal concentrations *p*. At percolation threshold $p=p_{c}$, continuous gold channel spans the system; (**b**) experimental images of the localized optical excitation in a gold/glass composite at percolation threshold ($p=p_{c}$). These images are collected with a SNOM using an excitation wavelength of λ=780nm, reprinted with permission from [76], Copyright 2007, World Scientific Publishing Co. Pte. Ltd.

**Figure 4:**
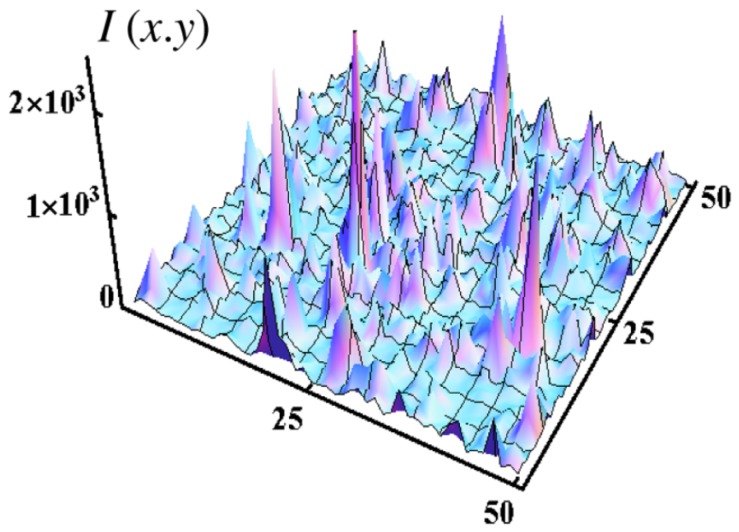
Collective volume plasmons in manganite ${\mathrm{La}}_{0.7}{\mathrm{Ca}}_{0.3}{\mathrm{MnO}}_{3}$ with nanoscale phase separation; computer simulation of infrared electric field $I={\left|E\left(x,y\right)/E_{0}\right|}^{2}$ at volume concentration $p=0.28>p_{c}$ of conducting phase; local permittivity ε(r) is positive in the dielectric phase Re[ε(r)]>0 and it is negative in conducting phase Re[ε(r)]<0, reprinted figure with permission from [81]; Copyright 2006 by the American Physical Society.

Giant electric field fluctuations and the related enhancement of nonlinear optical phenomena in semicontinuous metallic films are an area of active studies. Random metal-dielectric films are generally carried out on a glass substrate (insulating substrate) with several evaporation techniques such as thermal evaporation or sputtering of metallic layer. The static conductivity of the gold/glass composite is decreased when the percent of metal is decreased. At a critical percent $p_{c}$ called percolation threshold, the composite undergoes a composition-dependent metal-insulator transition. The composite behaves as a dielectric below this threshold (Figure 3a). In a series of works, it was shown that at the percolation threshold fluctuations of the EM field reach the enormous values (Figure 3b and Figure 4) [76,77,78,79,80]. The local electric field strongly fluctuates in any nanocomposite, where the local permittivity fluctuates between negative and positive values (see Figure 4).

## 3. Surface Enhanced Raman Scattering (SERS)

The resonances present in dielectric or metal-dielectric micro/nanostructures allow the confinement of the electromagnetic field at the nanoscale, and thus they can potentially improve the enhancement of the Raman scattering [82,83,84]. The Raman scattering is a phenomena of inelastic scattering, in which the vibration modes of the bonds of a molecule modulate an incident optical field at a higher or lower frequency and thereby get imprinted onto it. Spectrally, it observes a central peak corresponding to the central carrier frequency and other peaks with higher frequencies corresponding to anti-Stokes shifts and lower frequencies corresponding to Stokes shifts. Therefore, Raman spectroscopy can probe the structural information of the given molecules thanks to the vibrational modes of the bonds which compose them. The main interests of the Raman spectroscopy are its working mode in visible domain instead of an infrared one, its utility for molecular sensing due to the abundance of both high intensity coherent radiation sources, as well as of sensitive detectors operating in the visible domain, but also a possible extraction of the molecular structure informations at a high spatial resolution as a result of a substantially lower diffraction limit at visible wavelengths [85]. Surface enhanced Raman scattering (SERS) is one major physical phenomena of the last quarter of the twentieth century. Nevertheless, Raman scattering has a very weak scattering cross section. Moreover, the Raman signal can be potentially hampered by the background luminescence [85,86,87,88,89,90,91,92,93,94,95,96,97].

**Figure 5:**
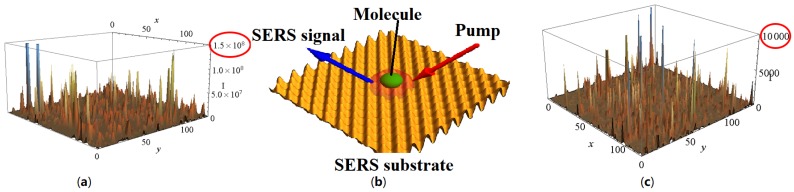
Schematic picture of SERS effect, see (**b**); incident light excites the collective plasmon field in semicontinuous metal film (see **c**), primary field); Raman active molecules, being pumped by the primary electric field, excite secondary EM field at the Stokes shifted frequency, see (**a**).

The generation of several plasmon modes on a metallic surface and further Raman scattering of the plasmons by analyte molecules are the basis of the SERS sensing (Figure 5). The molecules excited by the incident light and the plasmons can generate secondary plasmons which can be significantly enhanced. The radiation coming from these secondary plasmons produces a SERS signal [76,77]. Thus, the intensity of SERS signal is depending on the fourth power of local enhancement of the incident electric field. Nano- and micro-structures act as antennae efficiently amplifying the Raman signal. Several groups have already demonstrated enhancement factors from $10^{4}$ to $10^{9}$ for SERS substrates composed of clusters of gold or silver nanoparticles encapsulated in a dielectric matrix [98,99,100,101,102,103,104,105,106,107,108,109]. Moreover, a record enhancement up to $10^{12}$ of the Raman signal was reported by some authors [110]. It should be noted that a main contribution to SERS is the electromagnetic enhancement. The chemical enhancement strongly depends on local electronic structures of the molecules and the substrate it interacts with as each of their wavefunctions begin to overlap [111,112,113]. Some groups have demonstrated a chemical enhancement of SERS but with significant difficulties. Moreover, its influence is significantly weaker than electromagnetic enhancement. Indeed, the magnitude order of the chemical enhancement is only $10^{2}$ [114,115]. Thus, SERS effect (electromagnetic enhancement) allows detecting a weak concentration of biological and chemical molecules. However, in spite of all the efforts by many bright researchers, there is no self-consistent theory of the SERS effect. For instance, contemporary theories do not explain why the enhancement is so different for various Raman spectral lines as it is clearly seen in the next section. The authors heard the following opinion from the people dealing with SERS: “The surface enhances what ever it wants”. More recently, new highly sensitive SERS substrates have been carried out by using semiconducting materials (silicon or zinc oxide) with a metal (Au, Ag, Al). A couple of groups already demonstrated higher SERS enhancements obtained with Si nanowires coupled to metallic nanoparticles [116,117,118] and Si nanopillars coupled to a metallic layer [119,120,121,122]. Furthermore, other groups also demonstrated the same thing with ZnO nanowires/nanopillars coupled to a metallic layer [20,123,124] or metallic nanoparticles [125,126,127].

SERS phenomenon is also used in clinical diagnostics, which include the cancer detection and imaging, cancer therapy and drug delivery [128,129,130,131,132,133,134,135,136,137,138,139,140,141,142,143,144,145,146,147,148,149,150,151,152,153,154,155,156,157]. Important application of the SERS sensing is quantitative control of glycated proteins for diabetes detection [75,158,159,160,161]. Another very important SERS application is detection of the cardiovascular biomarkers for early diagnosis of acute myocardial infarction [162,163,164]. SERS detection of hormone Estradiol E2 is used for the clinical diagnosis of precocious puberty [165]. Environmental and food safety issues can be resolved using SERS real-time monitoring of pathogenic bacteria, pesticides and toxic molecules [166,167,168,169,170,171,172,173]. Ultra-low quantities of nerve gases, explosive substances and other hazard substances are also detected [174,175].

## 4. Field Enhancement in Dielectric Resonators

To illustrate SERS effect, we consider the EM field enhancement in the dielectric, transparent layer that is placed on a metallic substrate. The EM field for the incident light excitation, which propagates along a “*z*” axis normal to the layer, can be written as $E=E_{0}\exp\left(ikz\right)$, where k=ω/c=2π/λ is the wave-vector, λ is the wavelength. The electric field on the surface of the dielectric layer equals:

(4) $$E_{\mathrm{sur}}=2E_{0}\exp\left(-idk\right)/\left[1+in\cot(ndk)\right],$$

where *n* denotes the refractive index of the dielectric layer. The surface field achieves the maximum $E_{s}=2E_{0}$, where $E_{0}$ when the layer thickness d=(2m+1)λ/4n,m=1,2,…. The surface EM field amplitude is larger than the incident field by factor 2. The enhancement of the Raman scattering is given by:

(5) $$G=\frac{\langle {\left|E_{\omega }\left(r\right)\right|}^{2}{\left|E_{\omega -\Delta \omega }\left(r\right)\right|}^{2}\rangle }{{\left|E_{0}\right|}^{4}}≃\frac{\langle {\left|E_{\omega }\left(r\right)\right|}^{4}\rangle }{{\left|E_{0}\right|}^{4}},$$

where ω is the excitation frequency, Δω corresponds to the Stokes shift of the frequency; the second equation holds for Δω≪ω (see Refs. [76,81,176]). From Equations (4) and (5), the enhancement achieves its maximum value of G=16. Therefore, a simple dielectric layer with a thickness of a few hundreds of nanometers increases the Raman signal more than an order of magnitude.

**Figure 6:**
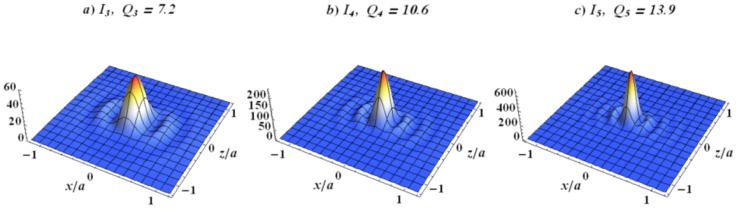
Electric field distribution $I_{m}={\left|E/E_{0}\right|}^{2}$ and $Q_{m}$ factor for a ceria $\left({\mathrm{CeO}}_{2}\right)$ spherical particle (a=400nm). (**a**) $m=3,\;\lambda_{3}=716\;$nm; (**b**) $m=4,\;\lambda_{4}=507\;$nm; (**c**) $m=5,\;\lambda_{5}=393\;$nm. All the parameters are available in [16], reprinted with permission from [16], the Optical Society (OSA).

The excitation of plasmon and dielectric resonances can enhance the Raman signal. From the hypothesis that the resonance frequency equals $\omega_{m}$ and the resonance width is higher than the Stokes shift of Δω. Moreover, from Equation (5), the effective SERS is obtained when the frequency of the excitation laser ω is within the interval is $\omega_{m}-\Delta \omega <\omega <\omega_{m}$. McFarland et al. were the first to obtain experimentally these types of results [98]. For all the dielectric nanoparticles, both EM resonances can be excited, for instance, with ceria, silica, and other dielectric materials [60,61]. Explosive molecules are detected thanks to a semiconducting resonator that is part of a plasmonic laser [177]. The EM field enhancement for the WGM resonators can be employed for the detection of biological and chemical molecules [178]. The latter allowed for detecting different particles with several sizes [179], batteries or viruses [59], and single molecules [4]. The EM field for a dielectric resonator can be confined in a hotspot. For example, a simple dipolar Mie resonance excited in the spherical ceria particle is displayed in Figure 6. Recently, several investigations have been made for obtaining the refractive index of ceria [180,181,182,183,184,185,186,187]. The refractive index *n* of ceria is depending on the wavelength, structure of the films [182,183,186], temperature [181,185], and the RF power employed for the magnetron sputtering of the film deposition [187]. The refractive index is higher with the denser ceria films. Thus, for the monocrystal particles, the refractive index of ceria is n≃2.3 is discussed below.

**Figure 7:**
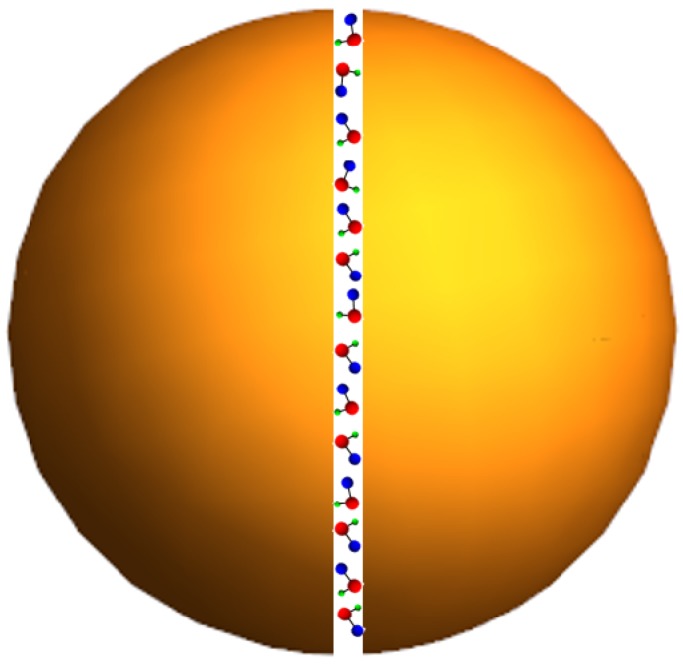
Slotted spherical dielectric resonator, reprinted with permission from [16], the Optical Society (OSA).

When the dipolar mode is excited, the EM field is enhanced at the center (see Figure 6). The highest value of the EM field is estimated in the following manner. The energy outflow from an eigenmode is approximated as $S_{e}∼a^{2}{\left|E_{0}\right|}^{2}$, where *a* corresponds to the dielectric sphere radius, and $E_{0}$ corresponds to the surface electric field. The field intensity $I_{m}={\left|E_{m}\right|}^{2}$ concentrates in the center of the particle for the dipole eigenmode (Figure 6). The radius of the field maximum is estimated as $r_{m}∼a/m$, where *m* in the order of the dipole resonance. The energy outflow $S_{m}$ from the maximum is $S_{m}∼r_{m}^{2}I_{m}$. By equating the energy flows $S_{e}$ and $S_{m}$, we obtain $I_{m}∼E_{0}^{2}m^{2}$. The highest value of the electric field is $m^{2}$ times elevated than the outside field as displayed in Figure 6. This result holds when the dissipate loss is much smaller than the radiation loss as discussed in [60,188]. The eigenfrequency, as well as electric field mapping in the electric dipolar resonance of Mie for an excited dielectric sphere, can be found analytically (see, e.g., [189]). When calculating the eigenmode, the radiation boundary conditions are imposed. That is, the outgoing wave propagating outside the sphere is assumed. The EM field is decaying due to the radiation loss; therefore, the eigenfrequency of any EM mode is a complex value $\omega_{m}=\omega_{m}^{\prime }-i\omega_{m}^{\prime \prime }$. We obtain the quality factor for the dipole resonances:

(6) $$Q_{m}=\frac{\omega_{m}^{\prime }}{2\omega_{m}^{\prime \prime }}\approx \frac{\pi \left(2\pi^{2}m^{3}-\pi^{2}m^{2}-2m-1\right)}{4\left[(\pi^{2}m^{2}+1)\mathrm{arccoth}\;n+n\right]}≃\frac{\pi }{4}\left(2m-1\right)\;n,$$

where *m* is the radial number (see Figure 6) and *n* is the refractive index [16]. This simple equation holds for n>1 and m>n. Thus, a significant confinement can be achieved by using a dielectric structure. Now, the molecules to be studied are placed in the slit as depicted in Figure 7. Next, the Raman enhancement *G* proportional to $\langle {\left|E\right|}^{4}\rangle$ can be evaluated as $G∼I_{m}^{2}E_{0}^{-4}∼Q^{4}∼{\left(mn\right)}^{4}\gg 1$. Thus, the SERS enhancement for dielectric structures could be even important than this obtained for plasmonic structures.

In addition, the electric field can be enhanced for dielectric structures for certain characteristic frequencies as depicted in Figure 6. For the planar dielectric metamaterials, discussed in the next sections, the EM field can also be confined in gaps between each dielectric structure (cf., Figure 2 and Figure 7). The Raman signal has few characteristic Stokes shifts $\Delta \omega_{i}\ll \omega$ [190]. When the set of dielectric resonances coincides with the set of Raman spectral lines, the dielectric resonator can be used as a sensor for the particular molecule. Both closely packed dielectric resonances corresponding to resonance splitting can be organized as follows: two dielectric spheres separating a *d* center-to-center distance are considered. There are three independent dipole modes $p_{x},\;p_{y}$ and $p_{z}$. The interaction energy of dipoles is estimated as $\Delta U_{m}∼\left(p_{m}\cdot p_{m}\right)/d^{3}∼p_{m}^{2}/d^{3}$, where $p_{m}$ corresponds to the dipolar moment of the *m* eigenmode. The dipolar moment square estimates as $p_{m}^{2}∼\varepsilon^{2}I_{m}r_{m}^{6}$, where $I_{m}$ and $r_{m}∼a/m$ are the intensity and the radius of the *m*-th mode, respectively (see Figure 6). The ratio of interaction energy $\Delta U_{m}$ to the eigenmode energy $U_{m}=\varepsilon I_{m}r_{m}^{3}∼\varepsilon I_{m}{\left(a/m\right)}^{3}$ gives the frequency split $\Delta \omega_{m}/\omega_{m}∼\Delta U_{m}/U_{m}∼\varepsilon {\left(a/dm\right)}^{3}$. Mainly, the Stokes shifts are weaker than the excitation frequency of laser. Therefore, it is enough to have the gap between the spheres (2a − *d*) ∼a and excite the second order dipole modes to obtain the proper resonance shifts. Then, the cluster of dielectric spheres can be used for the SERS sensing of particular molecules. EM resonances in a dielectric lamp, whose symmetry is less than spherical symmetry, may have quasi-continuous spectrum (see Ref. [18]). Small clusters of metal particles are considered in [191]. The metallic nanoparticles arranged in a regular shape of pentagon demonstrate two resonances with a low *Q* which are separated by Δλ (Δλ>100 nm or $\Delta \omega >5\times 10^{3}$ cm${}^{-1}$). Thus, this type of plasmonic structure can be employed with difficulty for the application of SERS sensors.

**Figure 8:**
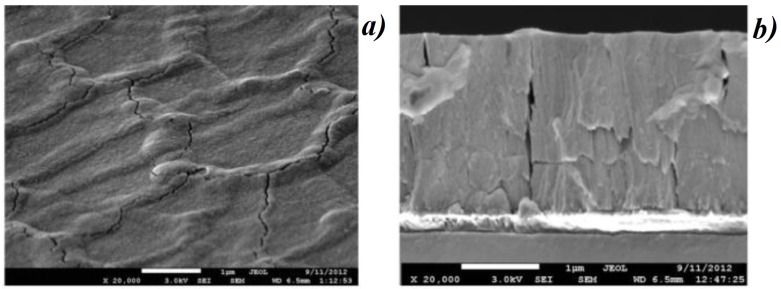
General (**a**) and cross-section (**b**) SEM views of ${\mathrm{CeO}}_{2}$ film that have facet structure; the facet structure is clearly seen: the facets are separated by cracks whose thickness is about ≃50 nm.

**Figure 9:**
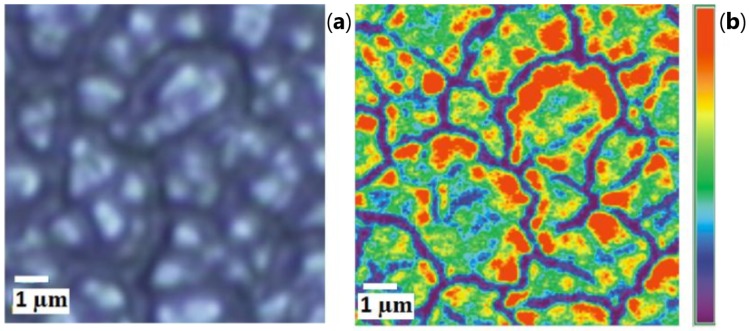
On (**a**), an optical image of 2400nm thick ${\mathrm{CeO}}_{2}$ surface is displayed, and on (**b**), an intensity distribution map for the main Raman peak, whose Stokes shift is of 1338 cm${}^{-1}$ from the laser frequency $12.738\times 10^{3}$ cm${}^{-1}$, i.e., wavelength λ=785 nm (CCD: red = 1700 counts, violet = 300 counts).

**Figure 10:**
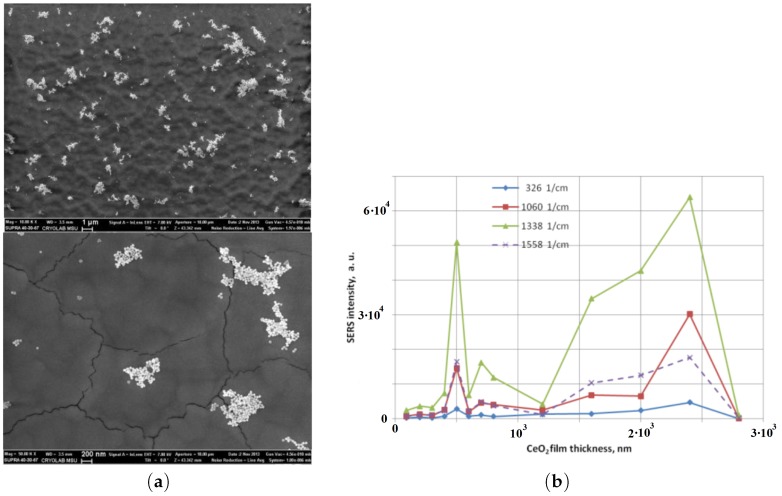
(**a**) SEM images of facet ${\mathrm{CeO}}_{2}$ films with gold nanoparticles (AuNP); scale bars are 1μm and 200nm for the top and bottom images, respectively; (**b**) SERS intensity for four Raman shifts from conjugate of DTNB/AuNP on ${\mathrm{CeO}}_{2}$ films as function of the film thickness (after normalization that is the Raman signal is divided into the number of AuNP in the spot, where the signal is collected from).

In [13], the authors have demonstrated the combination of plasmon resonances obtained with gold nanoparticles and localized EM resonances obtained with cerium dioxide films for a very sensitive detection of chemical and biological molecules by SERS. Morphology of cerium dioxide films is displayed in Figure 8. Nanostructures can form large clusters with a size of several hundred nanometers which are arranged in order to form structures with facets. The facet perimeter can correspond to a shape of curb. Each facet is spaced by small cracks of several tens of nanometers. Figure 9 depicts an optical image and a Raman intensity mapping for the Raman shift of 456 cm${}^{-1}$ of cerium dioxide. From these images, an irregular mapping of the signal on the film surface is demonstrated. The highest intensity of signal is generally located on the borders of facets. Toppgraphy of cerium dioxide films after deposition of gold nanoparticles (AuNP) is shown in Figure 10a. One to eight percent of the $\left({\mathrm{CeO}}_{2}\right)$ surface are occupied by the immobilized AuNPs. The non-regular mapping of the Raman signal on the $\left({\mathrm{CeO}}_{2}\right)$ film could indicate that a supplementary signal increasing is occurred after the immobilization of SERS tags on the facet surface. Raman signal was investigated from the conjugate of the DTNB and AuNP. The DTNB molecules (5,5${}^{\prime }$-Dithiobis-(2-nitrobenzoic acid)), also known as Elleman’s Reagent, bounded to the AuNP surface are the source of the Raman signal. Figure 10b depicts the SERS signal depending on the thickness of the $\left({\mathrm{CeO}}_{2}\right)$ film. The magnitude of the signal oscillates as a function of the film thickness. The additional enhancement for the SERS signal can be evaluated by normalizing this latter by the signal magnitude obtained with the film of 2800 nm thickness (minimal signal). For the Raman shift of 1060 cm${}^{-1}$, this additional enhancement is of a factor 200 times more important than with for the film of 2400 nm thickness. The SERS enhancement depends on the Raman shift studied. In a similar way, this means that the films with different thicknesses have a different selectivity relative to the vibrational modes having different frequencies. This important experimental result is in an obvious contradiction with the electromagnetic theory of SERS. All of the vibration modes of a molecule are excited by the same surface enhanced electric field. Therefore, the relative value of the Raman signals of various frequencies, i.e., various Stokes shifts depend on the Raman polarizability. The Raman polarizability is a molecule property and it should not depend on the ceria film thickness. Therefore, the relative value of the Raman signals with different Stokes shifts should not depend on the film thickness. In reality, we see in Figure 10b that the Raman signal from DTNB molecules with Stokes shift of 1558 cm${}^{-1}$ is larger than the signal with the Stokes shift of 1060 cm${}^{-1}$ in the film with the thickness from 1400 to 2100 nm. With further increasing of the thickness from 2100 nm to 2800 nm, the signal with Stokes shift of 1558 cm${}^{-1}$ becomes *smaller* than the signal with Stokes shift of 1060 cm${}^{-1}$. In addition, note that the Raman signal with Stokes shift of 1060 cm${}^{-1}$ is almost eight times smaller than this with a shift of 1338 cm${}^{-1}$ for the film thickness of 2000 nm. It is enough to slightly increase the film thickness from 2000 to 2400 nm and the ratio of these signals decreases from eight to two. It is not clear where such behavior of the SERS in the ceria facet film comes from. The experimental results cannot be explained using contemporary theories of SERS.

## 5. Metal-Dielectric Resonances

**Figure 11:**
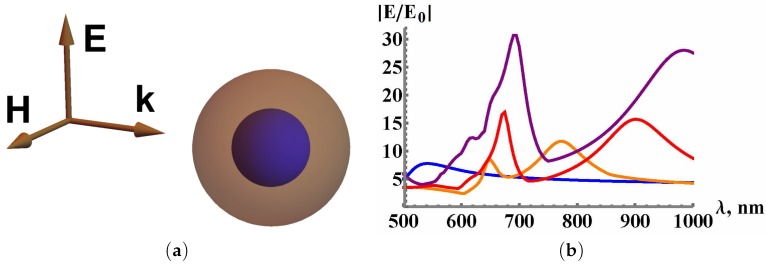
(**a**) scheme of the hybrid spherical resonator; (**b**) simulation electric field enhancement $|E_{\max}/E_{0}|$ in the resonator (gold core) for different thicknesses Δ of the dielectric shell. All the parameters are available in [18]. The blue line corresponds to isolated Au particle without dielectric shell and the orange, red and purple lines correspond to isolated Au particle with a dielectric shell of the thickness Δ=50nm, Δ=100nm and Δ=150 nm, respectively, reprinted with permission from [18], the Optical Society (OSA).

High EM field enhancements are mainly achieved with plasmonic resonators composed of gold (Au) or silver (Ag) nanoparticles. Metallic nanoparticles are well-known for having resonances thanks to the Faraday work (for recent references, see [1,2,76,192,193]). For a plasmonic sphere of which radius *a* is much smaller than the skin depth (i.e., $ak\sqrt{|\varepsilon_{m}|}\ll 1$), the electric field *E* behaves as $E_{in}≃3E_{0}/\left(\varepsilon_{m}+2\right)$, where $\varepsilon_{m}$ is the metal permittivity. From Ref. [50], it was demonstrated that an isolated gold nanoparticle presents a plasmon resonance at λ≃500 nm when this nano particle is excited in air. The resonance frequency can be tuned using a dielectric envelope. A hybrid (metal/dielectric) resonator which consists of a gold nanosphere with a radius *a* and a dielectric shell of thickness Δ is considered (see Figure 11).

The metal-dielectric resonator was considered using the hybridization approach (see Refs. [194,195] and references therein). The result of computational simulations of enhancement factor obtained with a hybrid resonator composed of gold nanoparticles (core) and a dielectric shell is displayed in Figure 11b. When Δ>a, the resonance of this resonator behaves almost a dipolar resonance of the dielectric sphere with a radius b=a+Δ. By using the asymptotic solution of Equation (7) referenced in the paper [16], the resonance of a dielectric sphere with the following parameters (a=200 nm and n=2.5) occurs at a wavelength of λ=690nm. This result is in good agreement with Figure 11b. The dielectric layer increases the internal electric field due to the existence of a plasmon-dielectric resonance. Several groups have investigated the effect of the cascade field enhancement for hybrid opto-plasmonic systems [16,19,20,65,66,196,197]. Santiago et al. have showed the detection of proteins with WGM resonators having hybrid (photonic/plasmonic) modes [198]. The *Q*-factor enhancement for hybrid (metal-dielectric) resonator is explained in the following way. The *Q*-factor increases in metal-dielectric resonators by decreasing the radiative losses. In Ref. [50], it was demonstrated for a resonator that the conducting current present in the metallic core and the polarization current present in the dielectric move with the opposite directions when the metal permittivity is mainly negative. The radiation is comparable to the sum of the currents, and the radiative losses decrease for a resonator, when the permittivities of resonator materials have different signs.

**Figure 12:**
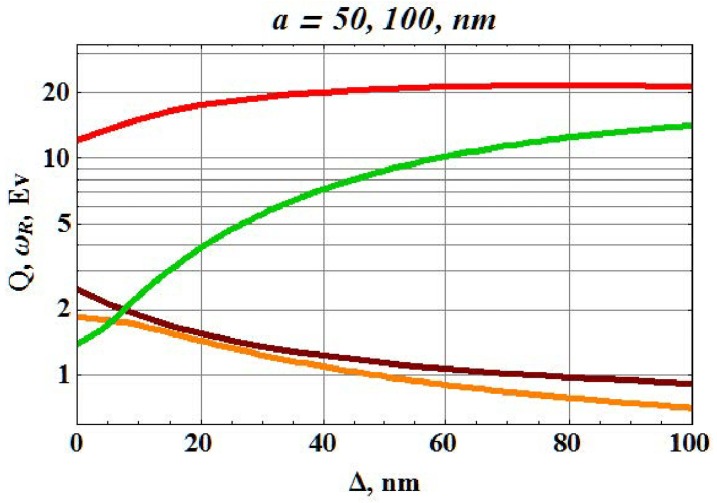
Resonance frequency $\omega_{r}$ (brown and orange curves) and *Q*-factor (red and green curves) of the gold-dielectric resonator, displayed in Figure 11, as function of the shell thickness Δ (refractive index of dielectric shell is n=4). Brown/red and orange/green curves correspond to the gold core radius a=50nm, and a=100nm, respectively, reprinted with permission from [18], the Optical Society (OSA).

In order to quantitatively examine the dielectric screening, the eigenstate of a plasmonic nanosphere with a dielectric shell (radius b=a+Δ>a, with *a* and Δ corresponding to the sphere radius and the dielectric thickness, respectively) is considered. Then, for simplicity, the electric dipole eigenstate is only studied. Matching the solution of the Maxwell equations in metallic and dielectric parts of the resonator with outgoing wave, we obtain a dispersion expression for an eigenfrequency $\omega =\omega^{\prime }-i\omega^{\prime \prime }$, which have an imaginary part due to the radiative losses. The resonator quality factor $Q=\omega^{\prime }/2\omega^{\prime \prime }$ can give the definition of the EM field enhancement in the resonator. A plasmon resonance in the gold nanoparticle of which the radius is *a*, is considered. For an approximate estimate, the Drude model can be used: $\varepsilon_{m}=\varepsilon_{b}-{\left(\omega_{p}/\omega \right)}^{2}/\left(1+\omega_{\tau }/\omega \right)$, where $\varepsilon_{b}=4.1,\;\omega_{p}=8.7\;\;\mathrm{eV},\;\omega_{\tau }=0.11\;\;\mathrm{eV}$ are chosen to correspond to the experiment [50] for ω<2eV. The *Q*-factor values of the gold nanoparticles without additional materials or layers (with the following parameters: $a_{1}=50\;$nm and $a_{2}=100\;$nm) are $Q_{1}\approx 12$ and $Q_{2}\approx 1.4$, respectively. This large difference of the *Q*-factor is due to the radiation loss when the radius was doubled. Indeed, the radiation is comparable to ${\left(ka\right)}^{3}$. When the metallic nanoparticle is surrounded by the dielectric layer, the radius of the metal-dielectric resonator increases. However, radiation losses decrease and the *Q*-factor increases, as displayed in Figure 12. We think that the radiation screening obtained by the dielectric layer improves *Q*-factor.

## 6. Metal-Dielectric SERS Metasurfaces

### 6.1. Periodic Bars

**Figure 13:**
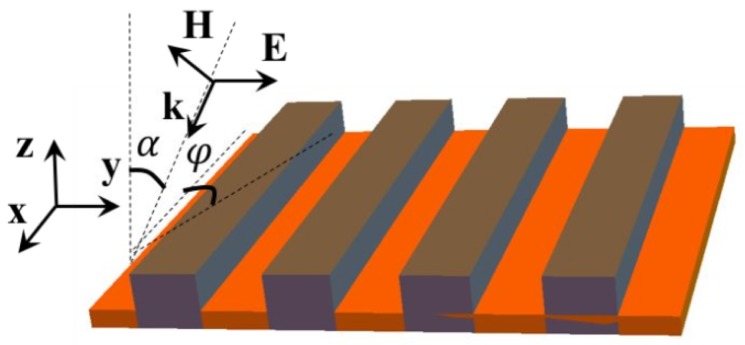
Scheme of the periodic polymethylmethacrylate (PMMA) bars on gold substrate, from [17].

**Figure 14:**
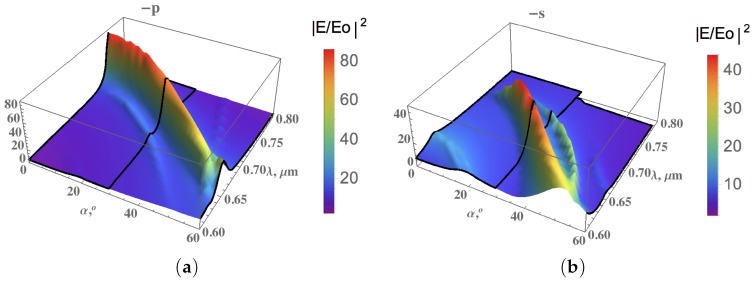
Intensity of electric field $|E/E_{0}{|}^{2}$ on top of PMMA bars for different wavelength λ and incidence angle α for (**a**) *p*-polarized wave and (**b**) *s*-polarized wave. The parameters are: an azimuthal angle of $\phi =2^{\circ }$, a period of L=635nm, a bar width of D=313nm, a bar height of $H_{\mathrm{PMMA}}=124\;$nm, a refractive index of PMMA: $n_{\mathrm{PMMA}}=n_{1}+in_{2}$ with $n_{1}=1.5$ and $n_{2}=0.001$, and the thickness of the gold substrate is $H_{\mathrm{Au}}=100\;$nm (from [17]).

Refs. [16,17] are devoted to the investigation of metamaterials of which the sharp resonances are separated by $\Delta \omega_{i}$ coming from metal-dielectric resonators. These types of metamaterials can be controlled in order to detect specific analytes with the signature $\Delta \omega_{i}$. A metal-dielectric composite metamaterial has been proposed. This metamaterial is composed of a silicon substrate on which is deposited a thick gold layer, then periodic bars of polymethylmethacrylate (PMMA) are fabricated (Figure 13). This system of periodic bars exhibits deep resonances in the wavelength range from 600nm to 800 nm for *p*- and *s*-polarized waves, where the EM field enhancement is significantly high as depicted in Figure 14. The main peak of the field intensity $|E/E_{0}{|}^{2}$ is on top of the PMMA bars. It shifts to a shorter wavelength with increasing the angle of incidence α. Moreover, the resonances for *s*- and *p*-polarized waves take place at different frequencies. It opens a new opportunity to tune SERS substrates for a particular analyte.

**Figure 15:**
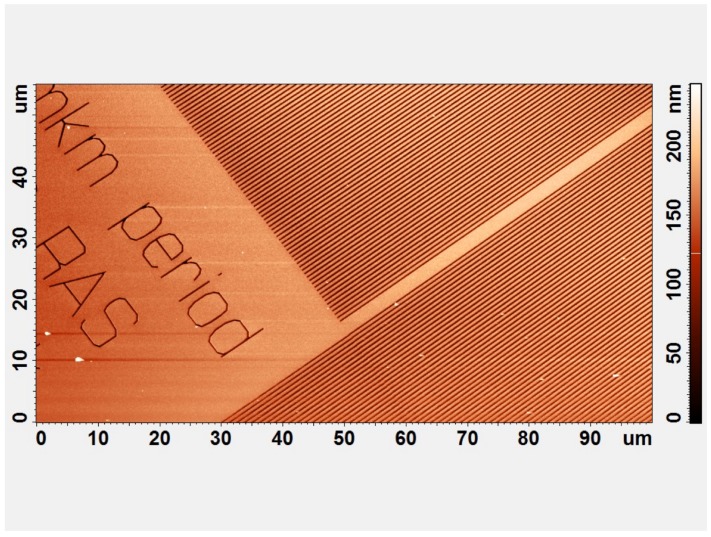
AFM of periodic dielectric structures based on Au and PMMA. The period equals 670–680 nm, gold thickness equals 40nm, reprinted with permission from [16], the Optical Society (OSA).

**Figure 16:**
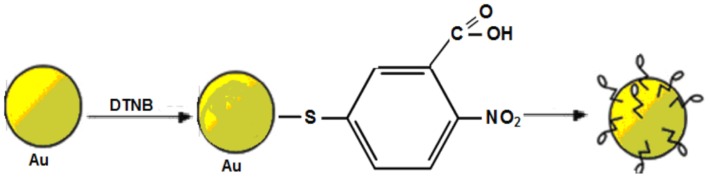
Principle scheme for preparation of AuNP-DTNB conjugate.

**Figure 17:**
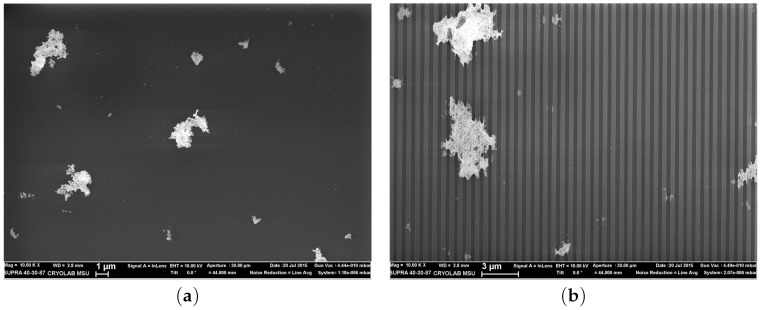
SEM images of AuNP-DTNB conjugates on: (**a**) flat and non-structured PMMA film; (**b**) PMMA bars; small bright spots are gold nanoparticles, some of them are organized in conglomerates, reprinted with permission from [16], the Optical Society (OSA).

The experimental realization of such SERS substrates based on PMMA was proposed in [16] to detect DTNB molecules immobilized on the surface as shown in Figure 15, Figure 16 and Figure 17. The metal-dielectric metasurface as described previously (Figure 15) was realized. Firstly, the sample size of 10×10×0.3 mm${}^{3}$ was cut from *n*-type phosphorus doped silicon wafer of which the resistivity is of 0.3 Ω·cm. After a chemical cleaning (in petroleum ether), the samples were cleaned with deionized water. Then, an adhesion layer of 4-nm Ti is evaporated followed by the deposition of a 40-nm gold film on Si substrate. Next, a PMMA thin film was spin-coated on the gold film in order to produce the PMMA film of thickness 600–1200nm. An electron beam lithography (Raith 150) has been employed for fabricating the nanobars on the sample surface. The dimensions of fabricated PMMA bars are 350-nm-wide lines with a periodicity of 700 nm on a $100\times 100\;\mu m^{2}$ surface area.

**Figure 18:**
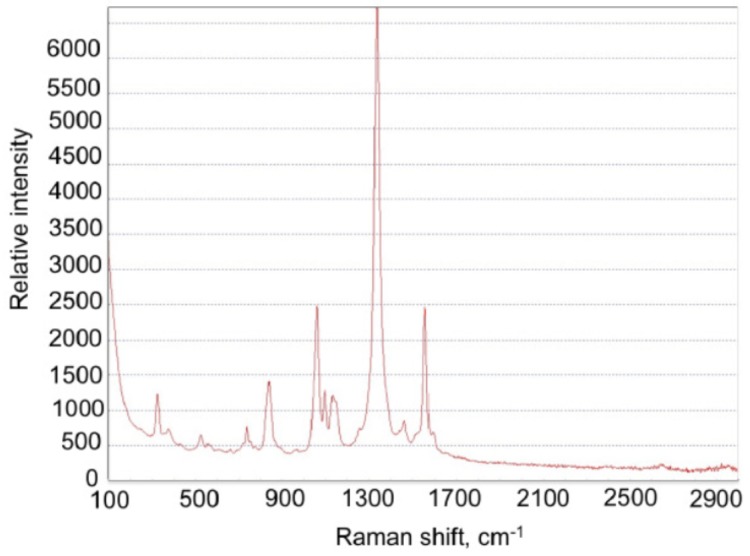
SERS intensity of AuNP-DTNB conjugates placed on PMMA bars (after normalization to the amount of gold nanoparticles as discussed in Figure 10), reprinted with permission from [16], the Optical Society (OSA).

To prepare SERS active particles, AuNPs of average size of 56±1 nm were modified by DTNB molecules as depicted in Figure 16. The conjugates of AuNPs and DTNB molecules were adsorbed to the bar-shaped metamaterial after a deposition of polyelectrolyte (poly(diallyldimethylammonium chloride)) with the process reported in the paper [199] (see Figure 17). The conjugate of AuNP with DTNB (AuNP-DTNB) has well-known Raman peaks and can serve as SERS indicator [200] (Figure 18).

The relative SERS intensity of AuNP-DTNB for the Raman shifts of 1338 and 1558 cm${}^{-1}$ was enhanced of a factor 5 for the bar-structured area compared to the flat PMMA layer. It should be noted that the Raman signal can be hindered by the background luminescence. The Raman/luminescence ratio (signal/noise) is most important for an effective SERS (for example, see Refs. [17,20,201]). We speculate that SERS substrates made of silicon look rather promising. Silicon has the advantage for SERS of having no luminescence background.

### 6.2. Periodic Blocks

**Figure 19:**
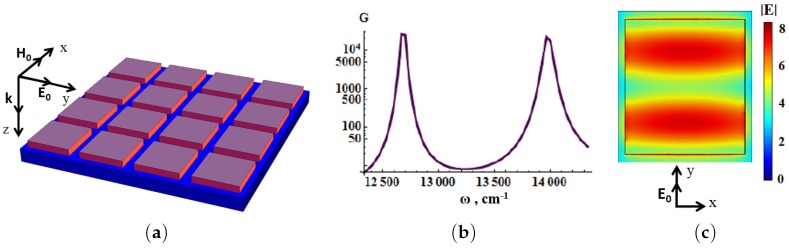
(**a**) 16 dielectric cuboids (purple) placed on the silver substrate (blue); (**b**) enhancement G as function of wavenumber for the dielectric cuboids placed on silver substrate, unit-cell dimensions of $D_{x}=650\;$nm, $D_{y}=550\;$nm, dielectric cuboid dimensions of dx = 585 nm, dy = 495 nm, and height h=55nm. The incident laser beam is normal to metasurface, electric field in the beam is directed along the diagonal of the dielectric blocks. The enhancement *G* is obtained at the surface of dielectric cuboid; (**c**) local electric field mapping on the top of dielectric cuboids of the first resonance shown in (**b**) (from [17]).

We believe the main drawback of the existing SERS substrates is an insufficient selectivity. In this section, we discuss high selective SERS substrates based on anisotropic periodic dielectric structures. We consider periodic dielectric metasurfaces and double periodic metal-dielectric metasurfaces, fabricated from dielectric cuboids made of ceria dioxide (${\mathrm{CeO}}_{2}$, *n* = 2.3). The cuboids are placed on silver and gold substrates (see Figure 19). Multiple plasmon resonances are excited near the metal-dielectric boundary as shown in Figure 19c. Figure 19b demonstrates that the Raman signal enhancement *G* has two maxima for the structure with different periods $D_{x}$ and $D_{y}$. The enhancement in the SERS substrate, shown in Figure 19a, has two peaks $\omega_{1}=12.77\times 10^{3}$ cm${}^{-1}$ and $\omega_{2}=13.95\times 10^{3}$ cm${}^{-1}$ with the widths of 200 cm${}^{-1}$ and 360 cm${}^{-1}$, correspondingly. The difference $\Delta \omega =\omega_{2}-\omega_{1}=1180$ cm${}^{-1}$ corresponds to the characteristic Stokes shift in the trinitrotoluene (TNT). This result was achieved by optimizing the dimensions of $D_{x},\;D_{y}$ of the unit-cell and dielectric cuboid dimensions of $d_{x},\;d_{y}$, and height *h*. The fabrication method of plasmon nanostructures consisting of dielectric blocks and highly sensitive SERS sensors was discussed in the previous subsection [13,202].

**Figure 20:**
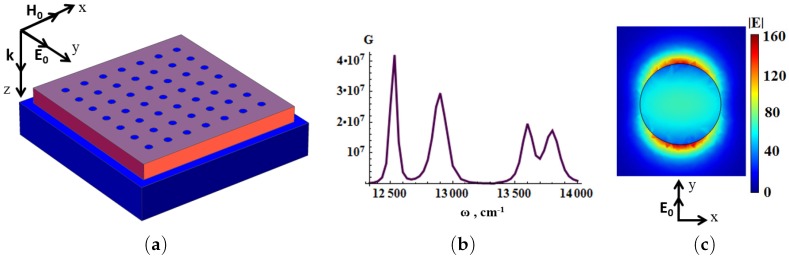
(**a**) silver nanodisks (small blue spots) deposited on dielectric (${\mathrm{CeO}}_{2},\;n=2.3$) cuboids (dark red) which are placed on silver substrate (blue); (**b**) Raman enhancement G as function of wavenumber; (**c**) distribution of local electric field $|E/E_{0}|$ around silver nanodisk with a wavelength of the incident light of λ=785nm. Geometric parameters: elementary sell sizes are $D_{x}=650$ nm and $D_{y}=550\;$nm (light blue in (**a**); cuboid dimensions are $d_{x}=585\;$nm, $d_{y}=495$ nm, and height h=47 nm (dark red), diameter of silver nanodisk $d_{c}=21$ nm and heght $h_{c}=3$ nm, aspect ratio of the nanodisk $h_{c}/d_{c}=1/7$, distance between nanodisks ≈60nm (from [17]).

To further increase the Raman enhancement *G*, a regular lattice of thin metal nanodisks was deposited on the top of dielectric cuboids, as shown in Figure 20.

**Figure 21:**
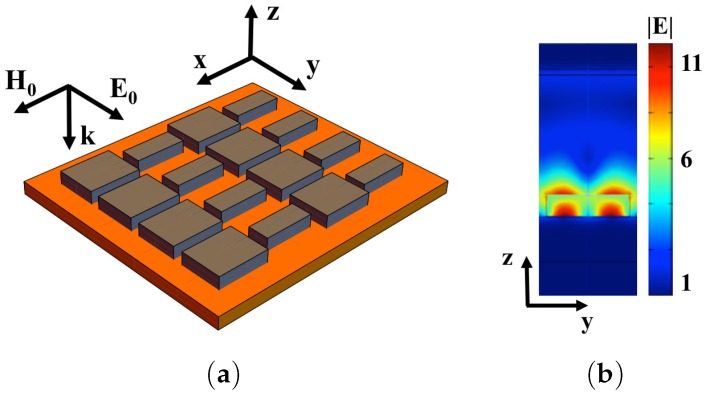
(**a**) 16 silicon dioxide blocks (eight elementary cells) placed on metallic substrate (orange). The metasurface has the following parameters: unit-cell dimensions of $D_{x}=1634\;$nm and $D_{y}=698\;$nm, dimensions of “large” dielectric blocks $d_{x1}=704\;$nm, $d_{y1}=568\;$nm, dimensions of “small” dielectric blocks $d_{y1}=704\;$nm, $d_{y2}=284\;$nm, height of the blocks $h_{1}=h_{2}=148\;$nm [16]; (**b**) electric field distribution $|E/E_{0}|$ when the incident light is normal to the metasurface at the frequency $\omega_{1}=11.4\times 10^{3}\;{\mathrm{cm}}^{-1}=1.41\;\mathrm{eV}$ (from [17]).

**Figure 22:**
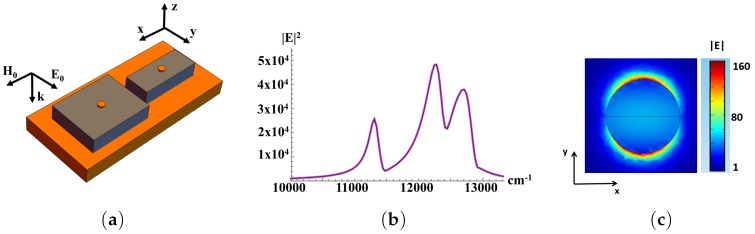
(**a**) single elementary cell of metal-dielectric substrate with gold nanodisks (orange color) placed on each of dielectric cuboids (gray), geometrical parameters are: lattice unit dimensions of $D_{x}$ = 1634 nm, $D_{y}$ = 698 nm, dimensions of dielectric resonators are $d_{x1}=d_{x2}=704$ nm and $d_{y1}$ = 568 nm, $d_{y2}$ = 284 nm, height of the resonators is *h* = 148 nm. The parameters of the top gold disk are $d_{c}$ = 50 nm, aspect ratio $h_{c}/d_{c}=\left(1/14\right)$; (**b**) electric field enhancement $|E/E_{0}{|}^{2}$ as function of wavenumber; gold disks are placed in the center of the top surface of dielectric cuboids. (**c**) electric field distribution $|E/E_{0}|$ over the gold disk. The metasurface is illuminated by light with an amplitude $E_{0}$ and a wavelength of 785 nm, the light is incident normal to the metasurface (from [17]).

Thereby, the cascade enhancement of the electric field is obtained. The resonating metallic nanoparticles are excited by the enhanced field of the composite metal-dielectric substrate (see Figure 20). The optimal surface concentration of the nanodisks equals $p_{m}\approx 7\%$. Silver nanodisks allow to increase the enhancement factor up to $10^{7}$–$10^{9}$ (see Figure 20). Note that decreasing of the distance between nanodisks results in the increase of dipole-dipole interaction and the peaks are dualized [203,204], see the discussion in the paragraph next to Equation (6). The surface morphology of SERS substrates can be more complicated. We consider the periodic planar dielectric structures of silicon dioxide (${\mathrm{SiO}}_{2},\;n=1.46$) dielectric blocks which are placed on a metallic substrate as shown in Figure 21. The chosen lattice is anisotropic with elementary cell dimensions of $D_{x}\times D_{y}$. The dielectric cuboids inside the elementary cell have dimensions of $d_{xi}$, $d_{yi}$ and the heights $h_{i}$. The resonance frequencies are tuned by the elementary cell design. Figure 21 demonstrates the high local electric field at the air-dielectric boundary. The SERS amplitude is given by Equation (5), where $E_{0}$ is the amplitude of the incident light. Figure 22 shows that the electric field enhancement has three maxima at three adjustment frequencies for the structure where the period $D_{x}>D_{y}$. The wavelength dependence of the local electric field intensity $|E/E_{0}{|}^{2}$ has three peaks, and can be tuned for two Raman spectral lines. The resonance frequencies are determined by independent variation of the periods $D_{x}$ and $D_{y}$, for $\left(D_{x}-D_{y}\right)<D_{x},D_{y}$. The difference between the peaks $\Delta \omega_{1}=\omega_{3}-\omega_{1}=1338$ cm${}^{-1}$, $\Delta \omega_{2}=\omega_{3}-\omega_{2}=326$ cm${}^{-1}$ ($\omega_{1}$ = $11.40\times 10^{3}$ cm${}^{-1}$, $\omega_{2}$ = $12.412\times 10^{3}$ cm${}^{-1}$, $\omega_{3}$ = $12.738\times 10^{3}$ cm${}^{-1}$) are tuned in order to correspond to the Stocks shifts of DTNB. Therefore, the discussed simple structure can be used for DTNB sensing. The enhancement of SERS signal can be additionally increased by combining plasmonic and dielectric resonators [16]. Thin metal nanodisks with diameter $d_{c}$ and height $h_{c}$ were inserted into the surface of the dielectric blocks (see Figure 22). We assume, for simplicity, that the nanodisks are made of the same material as the substrate. We vary the aspect ratio of the disks to tune the resonance frequency. As a result, the Raman enhancement $G∼|E/E_{0}{|}^{4}$ reaches the value of $10^{9}$ or even more for the substrate and nanodiscs made of silver (see Figure 22).

## 7. 3D Dielectric Resonators for Surface Field Enhancement

### 7.1. WGM Resonators

**Figure 23:**
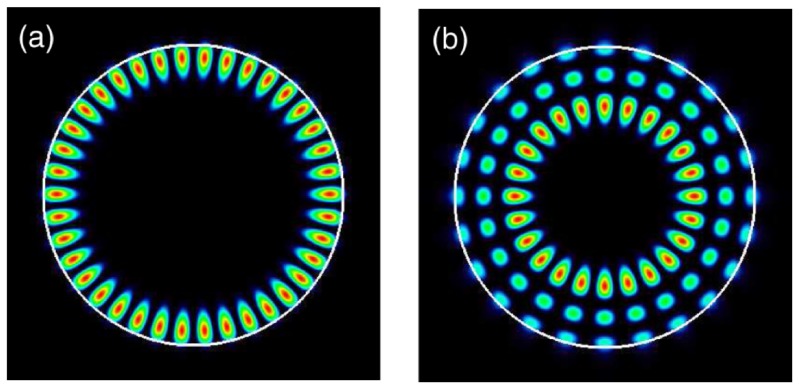
Whispering-gallery modes (WGMs) in a dielectric microdisk. The refractive index of GaAs is n=3.3, and the polarization is TM. The different numbers are (for more details (see [61])): (**a**) l=1 and m=19; (**b**) l=3 and m=12, reprinted figure with permission from [61], Copyright (2015) by the American Physical Society.

Large ohmic losses occur in metals that induce a damping of their optical response. Novel materials are to be developed in order to obtain good performances for the application to optical devices [205,206,207]. A promising way of development of such materials is based on dielectric materials [97]. A great number of EM modes exist, and the whispering-gallery modes (WGM) have the advantage of having large *Q*-factors. WGM has been a well-known phenomena through light interaction with dielectric interfaces for 100 years [3]. It is known in architectural acoustics that the sound propagates with a relative preference along the concave surfaces. In addition, the light can suffer a total internal reflection at the interface between a dense medium and a less dense medium for a certain angle of incidence. Thus, WGM can be understood as waves of the total internal reflection (Figure 23). The resonators based on WGM modes can be constituted of silica, ${\mathrm{CaF}}_{2}$, ${\mathrm{MgF}}_{2}$, GaN, GaAs, and have different shapes such as disk, torus, sphere or cylinder. The values of the *Q*-factors with such resonators can achieve $10^{7}$ and $10^{9}$ [4,53,54,55,56,57,208,209].

**Figure 24:**
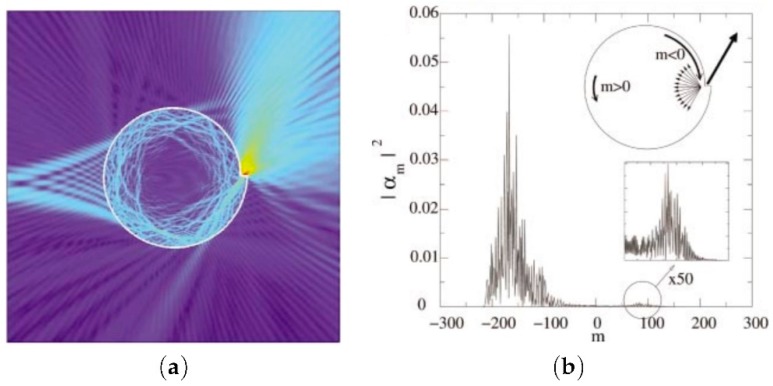
(**a**) real-space plot of the electric field modulus concerning to a calculated quasibound state at $nkr_{0}\approx 200$ with an eccentricity deformation (ϵ=0.10); (**b**) angular momenta distribution for the resonance plotted in (**a**). The peak at negative *m* corresponds to clockwise rotation, and the weak peak at positive *m* corresponds to counterclockwise rotation; the counterclockwise modes are the diffracted waves emitting from the notch. Reproduced from [213], with the permission of AIP Publishing.

Usually, the shape of the disk or sphere for resonators is employed. Some more complex 3D geometries exist and have supplementary degrees of freedom. Very interesting effects can be obtained with these complex geometries compared to simple ones. Sumetsky has demonstrated the light localization in a resonator with a shape of bottle named “whispering gallery bottle” [57,208,209]. Other shapes were investigated for dielectric resonators such as conical shapes [210,211,212]. In addition, a weak variation of the radius of an optical microcylinder implies strongly localized WGMs in the conical shape. The deformation of symmetry can lead to fascinating phenomena. In Ref. [213], an effect of unidirectional lasing with ${\mathrm{In}}_{0.09}{\mathrm{Ga}}_{0.91}N/{\mathrm{In}}_{0.01}{\mathrm{Ga}}_{0.99}N$ multiple-quantum-well spiral micropillars was demonstrated. The highest value of emission is achieved by the notch of spiral microcavity for an angle of about $40^{\circ }$ from the notch normal (Figure 24).

Thanks to their high *Q*-factor, WGM resonators can be employed for realizing filters, switches, lasers and sensors. In addition, several groups have reported the effect of quantum chaos for WGM resonators [61,214,215,216]. In addition, the nature and degree of the shape deformation can imply a change of whispering gallery orbits from regular shape to partially or fully chaotic. A long lifetime in a WGM resonator can enable the detection of single molecules or viruses onto the surface of this type of cavity [58,59] as mentioned above.

### 7.2. Cone-Shaped Resonator

**Figure 25:**
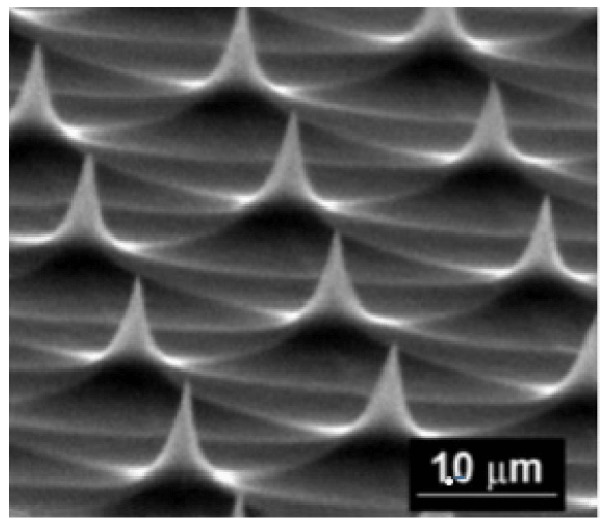
SEM of silicon tip-shaped metasurface, reprinted by permission from Springer Nature: Applied Physics A: Materials Science and Processing [217], Copyright 1998.

In Ref. [18], Lagarkov et al. have studied the light interaction with a tip-shaped metasurfaces composed of silicon cones (see Figure 25).

**Figure 26:**
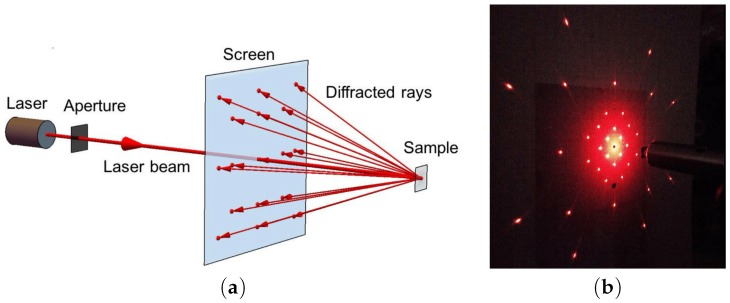
(**a**) principle scheme of the laser beam illuminating the metasurface for obtaining a diffraction pattern on the other side of the screen; (**b**) picture of this diffraction pattern obtained with a laser of wavelength λ=632.8nm. The other parameters are available in [18], reprinted with permission from [18], the Optical Society (OSA).

The geometrical parameters are: a square lattice period of w=2.1μm, a height of 0.3–0.7μm, the opening angle of the cone of $2\theta_{0}\approx 30^{\circ }$ a tip curvature radius of ≤10 nm, and a whole area of 2× 2 mm${}^{2}$. This metasurface composed of cones can be seen as a diffraction grating due to the fact that the inter-cone distance *d* is larger than the excitation wavelength λ in the visible domain. Indeed, the condition for obtaining a positive interference is that the difference in optical paths must be equivalent to an integer number of vacuum wavelengths. For obtaining a higher diffraction order, the necessary condition is that $-1<(m_{1}\lambda )/d<1$, $-1<(m_{2}\lambda )/d<1$, where $m_{1}$ and $m_{2}$ are diffraction orders. For instance, a HeNe laser (λ=632.8nm) (see Figure 26) is used, and 37 diffracting modes occurred (the case where $m_{1}=m_{2}=0$ corresponds to the reflected wave, and the cases $m_{1}=m_{2}=\pm 1,\pm 2,\pm 3$ correspond to the diffracted waves).

**Figure 27:**
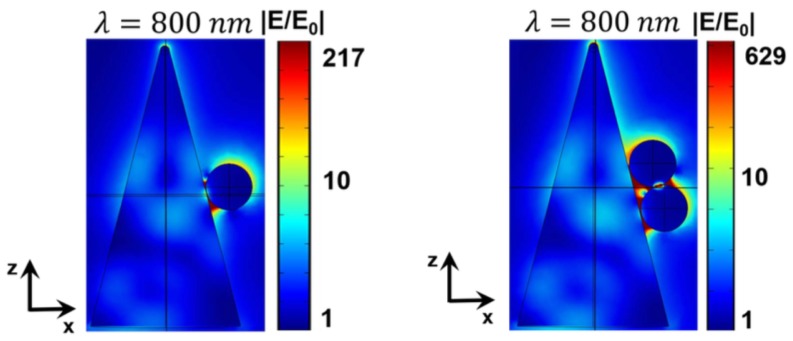
Electric field mapping $|E\left(z,x\right)/E_{0}|$ for Au nanoparticles (on the left, for one NP and, on the right, for two NPs, sphere diameter = 100 nm) placed on the lateral surface of a silicon cone for the resonant wavelength λ=800nm, reprinted with permission from [18], the Optical Society (OSA).

The traditional approach is used in the experiment. A HeNe laser (λ = 632.8 nm) illuminates the sample (metasurface) and reflects from this sample by producing a diffraction pattern on the other side of the screen as displayed in Figure 26. The parameters and settings are available in [18]. The diffraction pattern contains all the 37 modes having different radiances $I(m_{1},m_{2})$ as shown in Figure 26. The total reflection ($R=R_{00}+\sum R_{m_{1}m_{2}}$) is about ≃0.32, where 0.26 corresponds to zero order reluctance and 0.06 to the diffraction. In another manner, 19% of the energy reflected by the metasurface (sample) is provided to the diffraction beams. Moreover, these Si tips occup only 8% of the total surface of the sample. Thus, the diffraction is very efficient that we could call an “extraordinary” optical diffraction. This experiment is also possible by using another wavelength as λ=405nm and the result also gave a bright diffraction pattern Figure 26, even with large losses in silicon for wavelengths (λ < 500 nm) (see [218]). This observation is in agreement with discussions realized in [18] on the fact that the enhancement of the electric field is independent optical losses. No change of the diffraction pattern occurs when the system is moved with respect to the laser beam. We can deduce that the periodicity and the cone shape are well-defined on the whole sample. The highest value of the enhancement is obtained from the resonance involving Si cone and metallic nanoparticles placed on its lateral surface as seen in Figure 27. Surface plasmons in metal nanoparticles interact with EM modes in the dielectric cone, which results in huge enhancement of the local electric field.

**Figure 28:**
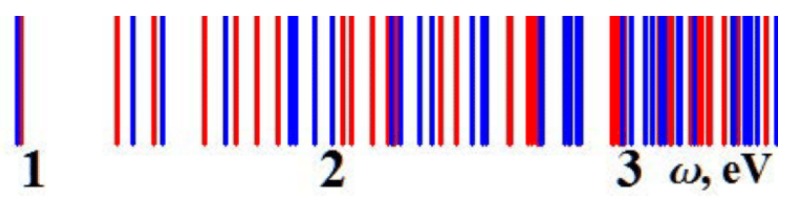
EM resonances spectrum for a conical resonator. The opening angle of cone is $30^{\circ }$, the refractive index is n=4, and the height is h=595nm. The red and blue lines correspond to frequencies of electric and magnetic resonances, respectively, reprinted with permission from [18], the Optical Society (OSA).

**Figure 29:**
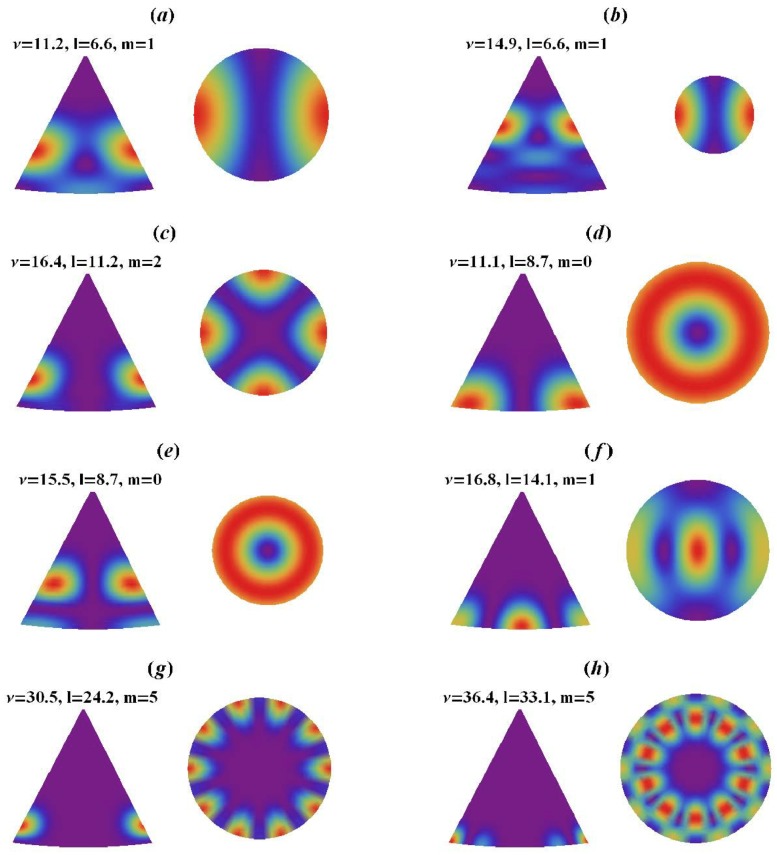
Electric field intensity ${\left|E\left(r,\theta ,\phi \right)\right|}^{2}$ in a dielectric conical resonator having an opening angle of $30^{\circ }$ and a refractive index of n≫1. “Electric” and “magnetic” resonances are depicted in Figure 28a,b,c,g and Figure 28d,e,f,h, respectively. Vertical distribution of field is determined by dimensionless frequency m=nkh, where *n*, k=ω/c, and *h* are refractive index, wavevector, and cone height, respectively. Each resonance is labeled by orbital “number’’ *l* and the azimuthal number *m*. The low symmetry of the resonator results in a non-integer *l*, reprinted with permission from [18], the Optical Society (OSA).

The EM field at the resonance is confined in a close vicinity to the cone. In addition, the interaction between each cone is negligible due to the distance between each cone of w=2.1μm, which is greatly longer than the cone size. Furthermore, the collective interaction can occur in the mid-IR frequency range (λ≃w). In this case, we think that collective surface modes can be excited. Lagarkov et al. have analyzed in a semi-quantitative manner the different resonances and the electric field enhancement for a dielectric resonator with a conical shape [18]. These dielectric resonators present several resonances in the visible and near-IR domains (see Figure 28). Due to an axial symmetry of the cone, the angular momentum is quantified and the modes with polar quantum number *l*, azimuthal quantum number *m*, and radial quantum number *q* are excited. The strongest value of the *Q*-factor corresponds to the WGM with the values of the numbers *l* and *q* which are minimal; however, the value of the number *m* is high (see Figure 29). The modes of which the values of polar and radial “quantum” numbers are weak propagate along the lateral surface of the cone. In addition, external modes also exist with weaker values of Q-factor where the electric field is partially located outside the cone. Moreover, the “leaky” region corresponds to these external modes with the needed condition that the total internal reflection is violated.

**Figure 30:**
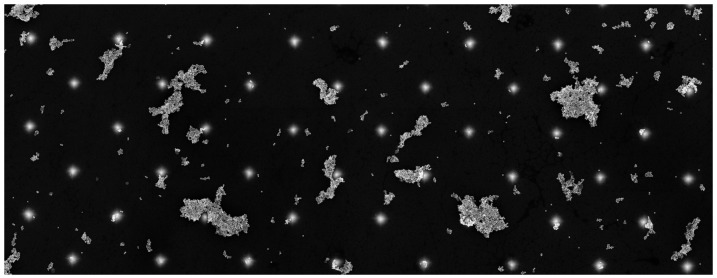
Aggregates of gold nanoparticles, which are seen as small bright white spots, are deposited on cone-shaped regular silicon metasurfaces; apexes of the cones are seen as a square lattice of 48 blurred specks, reprinted with permission from [18], the Optical Society (OSA).

**Figure 31:**
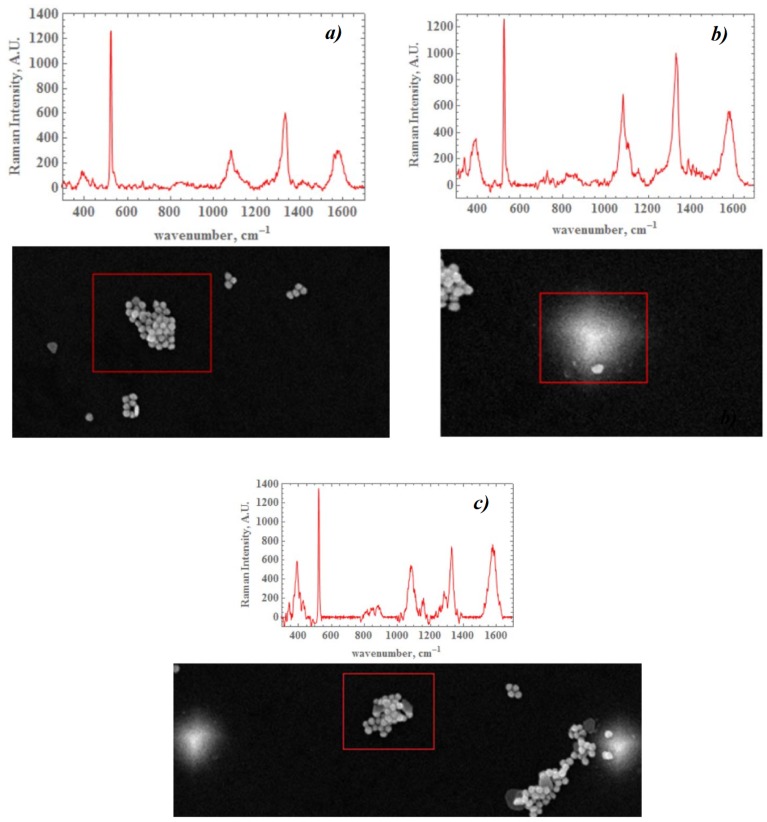
Raman intensity (SERS) obtained with AuNP-DTNB conjugates placed on different areas of cone-shaped metasurface; the areas where the Raman signal is collected are shown by red squares; apexes of the cones look like blurred specks, AuNP-DTNB conjugates look like bright spots. SEM images and SERS spectra recorded: (**a**) outside of grating, (**b**) on the pyramid side and (**c**) between pyramids. Reprinted with permission from [18], the Optical Society (OSA).

The metasurface composed of Si tips has been covered by SERS active tags consisting of Au nanoparticles of which the average size is 55±5nm, on which a DTNB monolayer has been deposited (see Figure 30). The DTNB molecules are grafted on gold nanopaticles thanks to their sulfate groups [13]. The Raman signal has been measured from AuNP–DTNB conjugate in order to have an approximate value of the EM field enhancement. We obtain a great enhancement of the intensity distribution for the metasurface compared to a flat region. This can be understood by excitation of the hybrid resonances (metal/dielectric) of Si cones covered by AuNPs. These conical Si tips serving as resonators enable converting the excitation light into longitudinal electric field. A detailed research has demonstrated that SERS intensity is depending on the position of gold nanoparticles on the surface (see Figure 31).

A strong SERS enhancement occurs when the gold nanoparticles are placed in a suitable manner on the lateral face of the cone which will induce hotspots. Thus, an electric field enhancement of more than three orders of magnitude can be achieved as displayed in Table 1. The light localization in this type of resonators (metasurface) opens the way to new possibilities in R and D for the fabrication of highly sensitive SERS substrates applied to biological and chemical sensing.

## 8. Local-Field Dielectric Transducer (LFDT)

**Figure 32:**
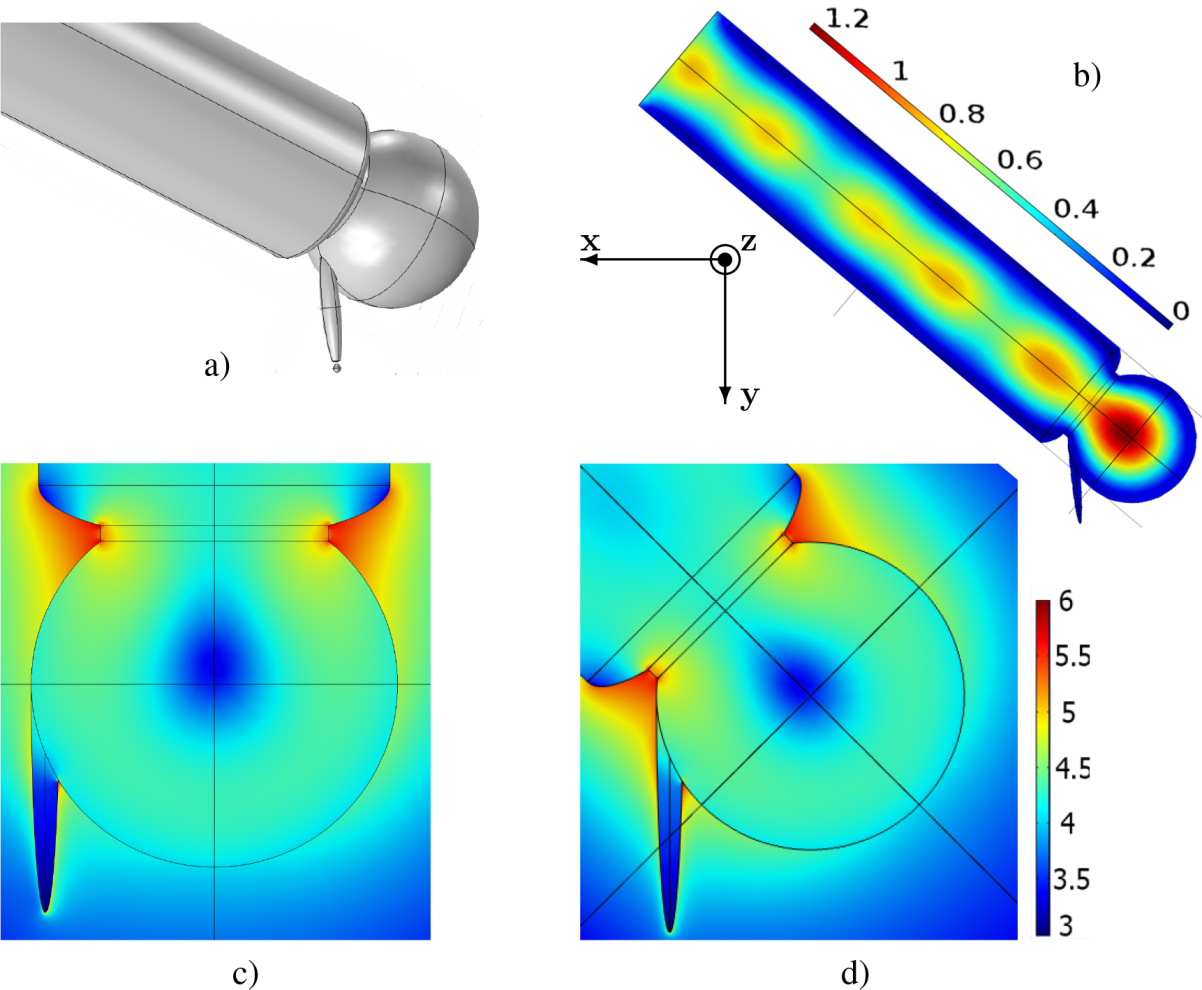
(**a**) scheme of a LFDT; (**b**) numerical simulations of the magnetic field |H|. Excitation of the stick by the electric field |E| with the following orientation of the waveguide: (**c**) vertical, and (**d**) inclined. All the parameters are available in [189], reprinted figure with permission from [189], Copyright (2017) by the American Physical Society.

Recent development of the plasmonics provides the possibility to concentrate the light onto a nano-area. Metal nanoantennae and subwavelength apertures [219] are used to better increase the local electric field in the subwavelength volume. The optical field enhancement and concentration are achieved by excitation of surface plasmons [220,221]. Nanosized metal particles are indispensable for most optical transducers and concentrators. Concentration of the huge electric field in a metal nanoparticle results in the fast degradation or even destruction of the particle [222]. To avoid negative effects of the large optic loss, it was proposed all-dielectric Local-Field Dielectric Transducer (LFDT), which enables confining of the light into a hotspot of nanometric size [189,223]. Negative thermal effects are nearly suppressed for LFDT allowing new possibilities in magnetic recording [224,225,226], optical sensing, and nanolaser pumping. LFDT is similar to waveguide gallery resonator with the notch, shown in Figure 24. The discussed LFDT is composed of a spherical dielectric resonator coupled to a dielectric nanostick, where the electric field is concentrated as shown in Figure 32. The EM field is stronger for the stick apex which is linked to the resonator. The field enhancement located at the stick apex was discussed by Novotny et al. [227]: the electric field aligned along the stick, periodically drives the bounded electrons. Electrons of an atom in the dielectric moves along the stick shaft with the same frequency as the excitation field. Around the apex, a large surface charge is present due to the small surface of apex and the uniform movement of bounded electrons. The accumulated charges gives the giant electric field (see Figure 32). With all the optical dense materials having small losses, an EM field enhancement can occur (see, e.g., [61,228]). The incident light is converted into a longitudinal electric field by means of the spherical resonator. Note that a simple waveguide cannot be used to effectively excite the longitudinal electric field [229]. Wang et al. have demonstrated the production of a longitudinal electric field with metallic LFDT composed of plasmonic lenses [230]. In the work [223], the dielectric stick is excited by using a waveguide connected to an optical resonator (Figure 32). This spherical resonator serves as an accumulator of the EM energy, and the energy stored in this latter excites the elliptical dielectric stick attached to the sphere. Note that the channelling of the whispering gallery modes was realized with an excellent efficiency in the connected waveguide with no supplementary loss (see Ref. [231]).

**Figure 33:**
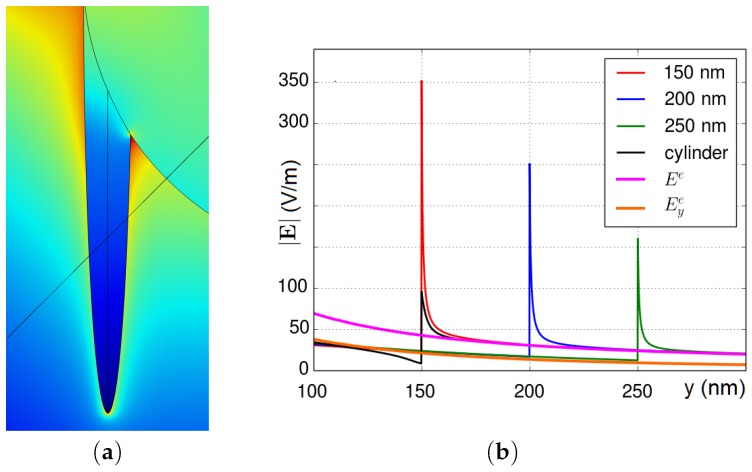
(**a**) scheme of a dielectric elliptical stick. The different parameters are available in [189]; (**b**) $|E^{b}\left(y\right)|$ corresponds to the field along the stick shaft for several lengths *b*. External $E^{e}$-field and its tangent projection $E_{y}^{e}$ in the absence of the stick are also displayed. Field peaks shows that the electrical field is confined in the nanovolume near the apex of the stick, reprinted figure with permission from [189]. Copyright (2017) by the American Physical Society.

**Figure 34:**
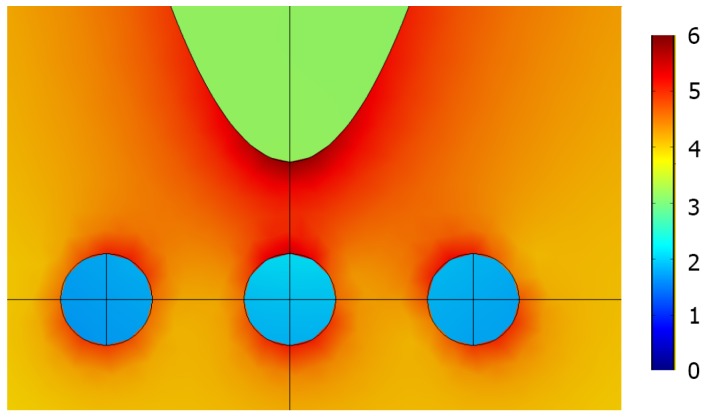
Tip heats magnetic nanoparticles (2nm) made of FePt alloy [232]. For the magnetic particle placed perfectly under the tip, the heat production is 1.4 times greater than the other neighboring particles, reprinted figure with permission from [189]. Copyright (2017) by the American Physical Society.

The Si waveguide with a cylindrical shape is connected to the resonator as depicted in Figure 32. As displayed in Figure 32, the stick is located at the interface between the resonator and the waveguide or opposite to the waveguide.The electric field is stronger for the inclined configuration of the stick. We think that for this configuration the field is more confined at the interface between the resonator and the waveguide. The field enhancement at the stick surface is shown in Figure 33. The field distribution was simulated by using three FePt nanospheres with a diameter of 2nm located within in the apex’s vicinity of the stick (see Figure 34). For magnetic recording assisted by heat, FePt nanoparticles are mainly employed [219,232].

The LFDT can be used for local sensing of various chemical and biological objects. There is a fascinating possibility to “illuminate” the investigated object and then collect the Raman signal from the spot, whose size can be less than one nanometer.

**Figure 35:**
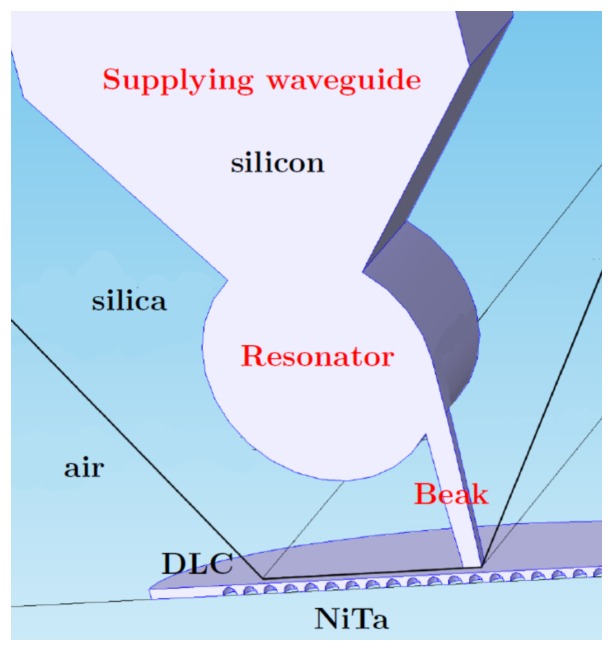
Scheme of a local-field dielectric transducer made of silicon. Diamond-like carbon (DLC) layer with equally spaced FePt grains is placed above NiTa substrate. FePt grains of size 14nm are arranged in the square lattice with the period of 20nm. The other parameters and informations can be obtained in Ref. [189]. Optical properties of FePt alloy and silicon were obtained from [232] and [233], respectively. The permittivity of NiTa alloy is calculated as the arithmetic mean of Ni and Ta permittivities. DLC permittivity is around $\varepsilon_{\mathrm{DLC}}=3.5$ coming from [234,235], reprinted figure with permission from [189]. Copyright (2017) by the American Physical Society.

To integrate the LFDT in the domain of electronics, the layer by layer growth can be more easily used, which is a well-known technique of the thin film technology. In order to decrease the number of the fabrication steps, a right cylindrical geometry of LFDT is adopted (in the direction of the growth which is perpendicular to the plane of Figure 35: *z*-direction).

**Figure 36:**
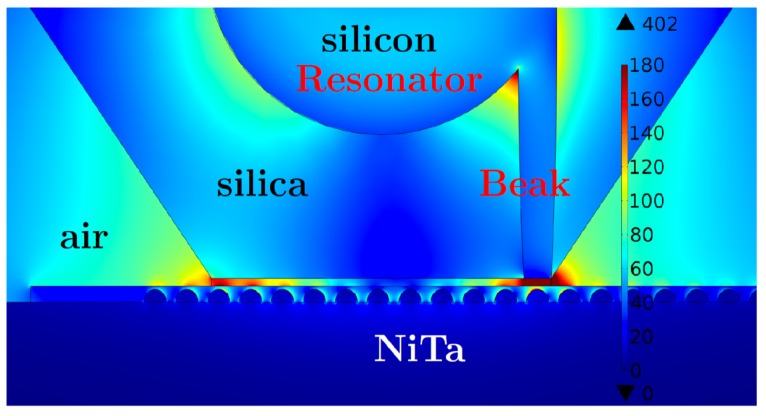
Electric field mapping below the LFDT, reprinted figure with permission from [189]. Copyright (2017) by the American Physical Society.

The disk resonator is considered. The waveguide and the stick have a rectangular shape. Several groups have demonstrated EM field confinements between two rectangular dielectric waveguides (see Refs. [236,237,238]), and other groups have studied a great number of disk resonators for RF applications for a couple of decades [239]. More recently, optical magneto-dipole resonances in silicon disks were demonstrated in Refs. [60,240]. The numerical simulations of 2.5-dimensional silicon LFDT are shown in Figure 36.

**Figure 37:**
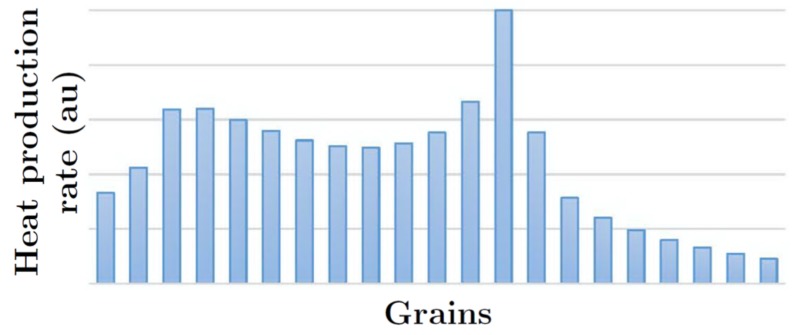
Heat production rate inside the grains in the central line. The highest heat production rate corresponds to the grain placed perfectly below the tip as displayed in Figure 36, reprinted figure with permission from [189]. Copyright (2017) by the American Physical Society.

The silica cladding incorporates the plane waveguide, the disk resonator, and the rectangular stick. The disk resonator and the plane waveguide have the same height (the size in the direction normal to the figure plane). The 2.5D LFDT is a solid-state device that can have a large size (macroscopic scale) and can be incorporated in present electronics. LFDTs can be used for the local sensing including the collection of the Raman signal from a single molecule. To demonstrate efficiency of 2.5D LFDT, the nanostructured substrate is considered. The substrate is a carbon matrix, where FePt nanoparticles are distributed [241,242]. The computer simulations give the heating of the Fe-Pt under the action of the LFDT as displayed in Figure 37.

When the tip is perfectly placed above a grain, the heat production is 1.6 times greater than in the case of neighboring grains. Thus, the 2.5D LFDT can be employed for the local detection of molecules with a spatial resolution of ∼10 nm (Figure 37). We think that the supplementary optimization could allow a resolution of ∼1 nm.

## 9. Conclusions

The low-loss and high-quality optical resonators are of great interest for the fundamental as well as applied research, as they are indefensible in optical filters, solar energy concentrator, medical imaging and biodetection including SERS, optical super-resolution microscopy, and magnetic recording assisted by heat, quantum electrodynamics and nanolasing. Optical resonators include regular or disordered plasmonic and dielectric micro- and nano-structures, which can serve optical antennae by concentrating large electromagnetic fields at micro- and nano-scales. The morphology of an optical resonator has an important key role in the control of its optical response. Thus, resonators with periodic structure allow for concentrating EM energy at any specified frequency. Such resonators can be used to reach additional SERS enhancements and increase the selectivity of SERS sensors. The approach based on “dielectric plasmonics” allows for fabricating high-*Q* light concentrators with low radiation losses. Dielectric hierarchical structures are designed in order to achieve a great field enhancement at the nanoscale. A great field confinement is obtained without losses. Thus, the EM field transducer is composed of a dielectric resonator and a stick having a sharp apex. The accumulation of the EM energy delivered by the waveguide is realized by the resonator. Therefore, the waveguide effectively pumps the resonator, which illuminates the stick and produces a strong electric field at the apex. Dielectric LFDTs arranged in an array can be employed as a SERS substrate without a luminescent background.

## Figures and Tables

**Table 1:** Raman intensity for the Raman shift of 1338 cm${}^{-1}$ of the conjugate AuNP-DTNB on the metasurface vs. flat plane (in a.u.). The signal is collected from red frames, shown in Figure 31; normalized signal is obtained by dividing by the number of Au nanoparticles in a frame.

Au-NP Localization	Signal (Counts)	Au-NP Number	Normalized Signal
Pyramid side	786	1	786
Between pyramids	553	30	18.4
Outside of grating	553	89	6.2

## Abbreviations

The following abbreviations are used in this manuscript:

AFM	Atomic force microscopy
ASNOM	Apertureless scanning near-field optical microscopy
AuNP	Gold nanoparticle
DLC	Diamond like carbon
DTNB	5,5${}^{\prime }$-Dithiobis-(2-nitrobenzoic acid)
EM	Electromagnetic
HAMR	Heat-assisted magnetic recording
LFDT	Local field dielectric transducer
PMMA	Polymethylmethacrylate
QED	Quantum electrodynamics
R & D	Research and development
SEM	Scanning electron microscopy
SERS	Surface-enhanced Raman scattering
SNOM	Scanning near-field optical microscopy
SP	Surface plasmon
TERS	Tip-enhanced Raman scattering
TNT	Trinitrotoluene
WGM	Whispering gallery mode
